# Risk stratification and prognostic value of multi-modal MRI-based radiomics for extranodal nasal-type NK/T-cell lymphoma

**DOI:** 10.1186/s12885-023-10557-3

**Published:** 2023-01-25

**Authors:** Yu-Ting Zhao, Si-Ye Chen, Xin Liu, Yong Yang, Bo Chen, Yong-Wen Song, Hui Fang, Jing Jin, Yue-Ping Liu, Hao Jing, Yuan Tang, Ning Li, Ning-Ning Lu, Shu-Lian Wang, Han Ouyang, Chen Hu, Jin Liu, Zhi Wang, Fan Chen, Lin Yin, Qiu-Zi Zhong, Kuo Men, Jian-Rong Dai, Shu-Nan Qi, Ye-Xiong Li

**Affiliations:** 1 State Key Laboratory of Molecular Oncology and Department of Radiation Oncology, National Cancer Center/National Clinical Research Center for Cancer/Cancer Hospital,, Chinese Academy of Medical Sciences (CAMS) and Peking Union Medical College (PUMC), Beijing, P. R. China; 2 Key Laboratory of Carcinogenesis and Translational Research (Ministry of Education/Beijing), Department of Radiation Oncology, Peking University Cancer Hospital & Institute, Beijing, P. R. China; 3 Department of Diagnostic Imaging, National Cancer Center/Cancer Hospital, Chinese Academy of Medical Sciences (CAMS) and Peking Union Medical College (PUMC), Beijing, P. R. China; 4 Division of Biostatistics and Bioinformatics, The Sidney Kimmel Comprehensive Cancer Center, Johns Hopkins University School of Medicine, Baltimore, MD 21205-2013 USA; 5 Blot Info & Tech (Beijing) Co. Ltd, Beijing, P. R. China; 6 Department of Radiation Oncology, Affiliated Hospital of Qinghai University, Qinghai, P. R. China; 7 Department of Radiation Oncology, Beijing Hospital, National Geriatric Medical Center, Beijing, P. R. China

**Keywords:** Extranodal nasal-type NK/T-cell lymphoma, Multi-modal magnetic resonance imaging, Radiomics, Prognosis

## Abstract

**Background:**

Magnetic resonance imaging (MRI) performs well in the locoregional assessment of extranodal nasal-type NK/T-cell lymphoma (ENKTCL). It’s important to assess the value of multi-modal MRI-based radiomics for estimating overall survival (OS) in patients with ENKTCL.

**Methods:**

Patients with ENKTCL in a prospectively cohort were systemically reviewed and all the pretreatment MRI were acquisitioned. An unsupervised spectral clustering method was used to identify risk groups of patients and radiomic features. A nomogram-revised risk index (NRI) plus MRI radiomics signature (NRI-M) was developed, and compared with the NRI.

**Results:**

The 2 distinct type I and II groups of the MRI radiomics signatures were identified. The 5-year OS rates between the type I and type II groups were 87.2% versus 67.3% (*P* = 0.002) in all patients, and 88.8% versus 69.2% (*P* = 0.003) in early-stage patients. The discrimination and calibration of the NRI-M for OS prediction demonstrated a better performance than that of either MRI radiomics or NRI, with a mean area under curve (AUC) of 0.748 and 0.717 for predicting the 5-year OS in all-stages and early-stage patients.

**Conclusions:**

The NRI-M model has good performance for predicting the prognosis of ENKTCL and may help design clinical trials and improve clinical decision making.

**Supplementary information:**

The online version contains supplementary material available at 10.1186/s12885-023-10557-3.

## Background

Extranodal nasal-type NK/T-cell lymphoma (ENKTCL) is a rare and aggressive neoplasm frequently seen in adults [1–3], usually manifested by involvement of the upper aerodigestive tract (UADT), such as the nasal cavity and Waldeyer ring [4–6]. ENKTCL is strongly associated with Epstein-Barr virus (EBV) infection and frequently presents with early-stage disease and primary tumor invasion (PTI) [4, 5]. Advances in upfront/early radiotherapy and non-anthracycline-based chemotherapy have improved the outcomes for patients with ENKTCL. Radiotherapy is the most efficacious modality and is an essential component of multidisciplinary management for early-stage ENKTCL [6–13], whereas asparaginase-based chemotherapy is the primary treatment for advanced-stage disease [14–16]. Several risk models from large collaborative studies have been developed to provide prognostic prediction for ENKTCL [5, 17–19]. More recently, the nomogram-revised risk index (NRI) [19], derived from the original visual nomogram model [5], has been shown to have excellent prognostic ability, and be effective in risk-adapted therapy for early-stage ENKTCL [7, 8]. However, optimization of risk stratification with incorporation of molecular or radiomics biomarker into the NRI warrants further investigation.

Radiomics could convert digital medical images into high-dimensional mineable data by using machine learning algorithm [20], and non-invasively extract the imaging features from tumors for the establishment of prognostic prediction models [21]. Previous studies have demonstrated that radiologic features and bio-imaging features derived from computed tomography (CT), magnetic resonance imaging (MRI) or positron emission tomography (PET) imaging provide the prognosis prediction in cancers [22–24]. MRI is most useful in ENKTCL because it provides good soft-tissue resolution of extension of the primary tumor into surrounding normal tissues and possesses multiplanar capability [25]. To define the primary site and PTI accurately, MRI is routinely recommended for optimal locoregional assessment for patients with ENTKCL, particularly UADT-ENTKCL [12, 25]. Thus, multi-feature signatures based on multi-modal MRI are assessable to investigate huge numbers of markers in a high-throughput manner. However, an association of MRI radiomics signature with survival has not yet been reported in patients with ENKTCL.

We hypothesized that incorporation of MRI radiomics signature into the previously well-established NRI model may be more effective and beneficial to predict outcomes for ENKTCL patients. In this study, we aimed to evaluate the prognostic effect of MRI radiomics in ENKTCL, and develop a combined model that integrates radiomics signature with clinical features for personalized prognostic prediction and treatment decision making.

## Methods

### Eligibility criteria

**Fig. 1:**
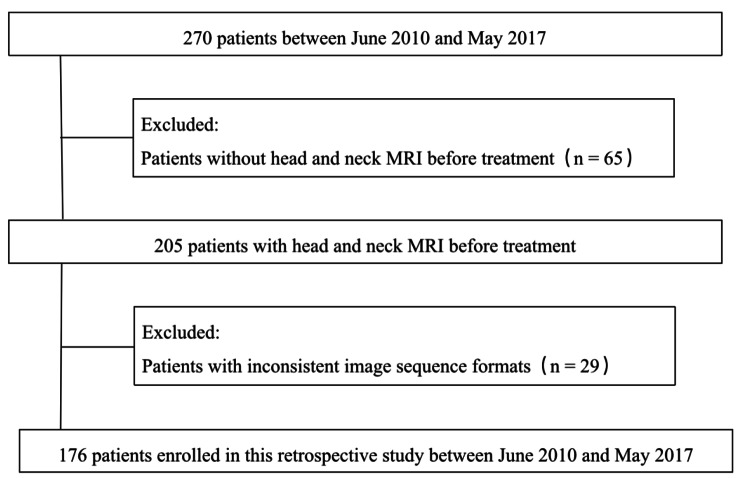
Patient selection flowchart

Patients with previously untreated ENKTCL between 2010 and 2017 were prospectively collected and systemically reviewed in a database from the National Cancer Center/Cancer Hospital, Chinese Academy of Medical Sciences (CAMS) and Peking Union Medical College (PUMC), Beijing, China. Eligibility requirements for this study were: (1) the typical histological and immunophenotypic features based on the World Health Organization classification; (2) primary tumor evaluable assessment by complete MRI scans with T1 weighted imaging (T1WI), fat-suppressed T2 weighted imaging (T2WI), dynamic contrast-enhanced T1-weighted imaging (DCEI) and diffusion-weighted imaging (DWI); (3) a comprehensive pretreatment staging evaluation; (4) patients primarily treated with non-anthracycline-based chemotherapy and modern radiotherapy. A total of 176 patients satisfied the eligible criteria were included in this study (Fig. 1). Our institutional ethics review board approved this study and patients signed the informed consent.

### MRI acquisition

All patients underwent T1 weighted, fat-suppressed T2 weighted, diffusion-weighted sequences and contrast-enhanced T1-weighted images. There are 4 MRI scanners used in our study. The MRI was performed using 3.0-T unit scanners (Discovery MR 750, GE Healthcare, Milwaukee, Wisconsin, USA) with eight-channel head and neck coil. The region from the top of the skull to the thoracic inlet was examined. Conventional sequences without enhancement were acquired with fast recovery of spin echo sequence, section thickness of 5 mm, intersection gap of 1 mm. Transverse T1WI was obtained with a repetition time (TR) of 660.0 ms, echo time (TE) of 9.3 ms. Transverse fat-suppressed T2WI was obtained with a TR of 5760.0 ms, TE of 88.3 ms. Contrast-enhanced T1WI were obtained with three-dimensional spoiled gradient recalled echo sequence after bolus injection of 0.2 ml/kg gadopentetate dimeglumine at 2ml/s. DWI was obtained with fast reverse recovery plane echo imaging sequence with a TR of 4500.0ms, TE of 89.0 ms, section thickness of 5 mm, intersection gap of 1 mm, field of view (FOV) of 24 cm×24 cm, matrix of 256 × 256, and 2 b-values (800 and 1000 s/mm2). On all slices, T1WI, T2WI, DWI and DCEI were exported as Digital Imaging and Communication in Medicine files. The tumor boundary was contoured and validated by two radiologists independently, while disagreements were further verified by a senior expert.

### Extraction of radiomics features

To normalize different image specifications due to the utilization of different MRI scanners, MRI signal-intensity normalization was performed. The Marching Cube (MC) algorithm was performed in our study, which is a voxel-level reconstruction method. We reconstructed two-dimensional data to three-dimensional data using this algorithm. The basic principle of the MC algorithm is to construct iso-surface in the three-dimensional data field. The voxel of the value surface is obtained, and the relevant parameters are calculated. The algorithm has a fast calculation speed and a high quality of the reconstructed image. The region of interest (ROI) was automatically extracted from T1WI, T2WI, DWI and DCEI. The AIMED was used for the feature extraction process (https://www.blothealth.com). For spatial resampling, three-dimensional reconstruction was performed, and ROI images were resampled to a voxel size of 1 × 1 × 1 mm, which could correct the pixel-spacing difference and restore the tumor volume. For intensity rescaling, the dcm data was read, and normalized with the means and standard deviations at pixel level. This step can reduce the difference between different data sets caused by equipment and make the model better fit to different data sets. The intensity was not discretized for the feature extraction process. A total of 3144 radiomics features were extracted from three-dimensional tumor volumes, with 540 histogram of oriented gradient features, 42 texture features, 48 wavelet features and 156 statistical features in each sequence. Normalization was also applied to the features for the sake of comparison, which was defined as follows:

1 $${x}^{*}=\frac{x-\sigma }{\mu }$$

### Selection and signature building of radiomics features

To build a realistic radiomics signature, the t-test (two-sided, alpha = 0.05) was used to remove the invalid features. Based on the selected features, a radiomics signature was developed using spectral clustering method. Spectral clustering is an unsupervised machine learning method based on data similarity matrix. It defines the optimization objective function of subgraph division, and makes improvements, introduces indicator variables, and transforms the division problem into solving the optimal indicator variable matrix H. Then, using the properties of Rayleigh entropy, the problem is further transformed into solving the k minimum eigenvalues of the Laplacian matrix. Finally, H is used as a certain expression of the sample, and clustering is performed using traditional clustering methods.

**Fig. 2:**
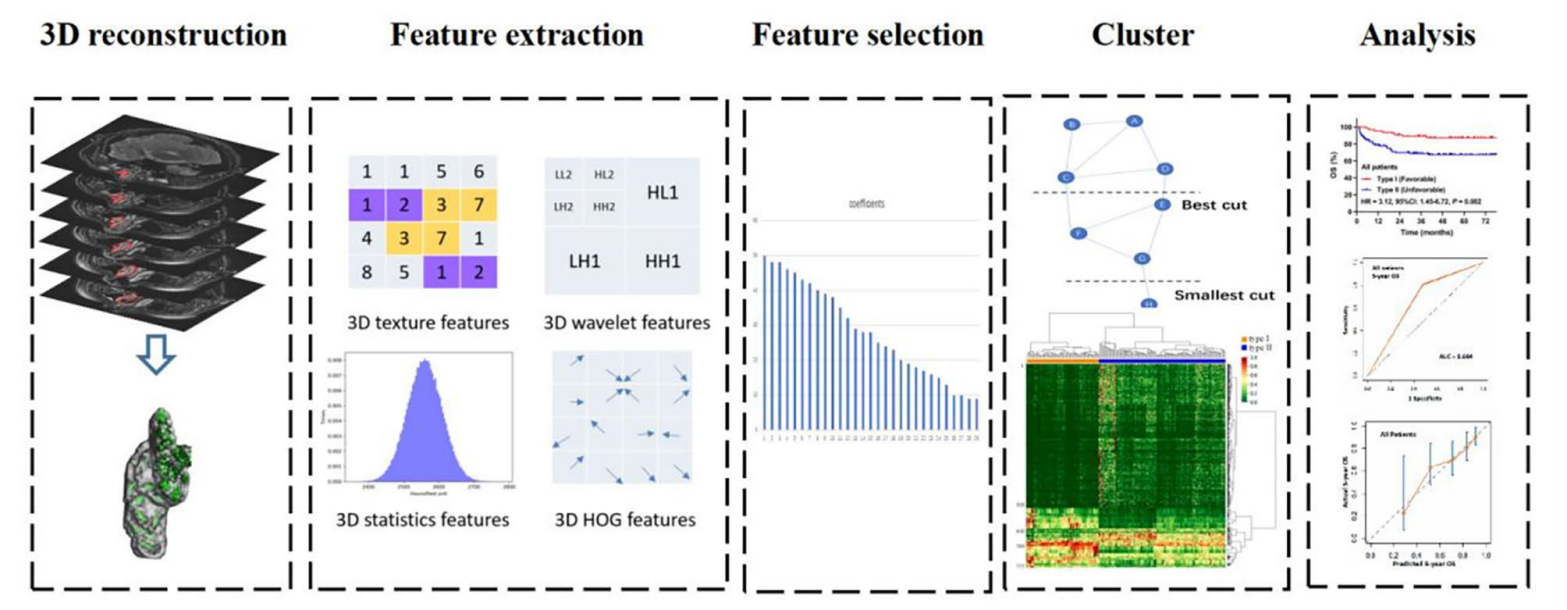
Workflow of necessary steps in the radiomics signature analysis. The ROI in each transverse section was segmented on T1-weighted, T2-weighted, diffusion-weighted and dynamic contrast-enhanced magnetic resonance images. After three-dimensional reconstruction of the ROI, features were extracted, and selected via t-test. Based on the selected features, a radiomics signature was developed using the spectral clustering method. The performance of the radiomics signature was evaluated with discrimination and calibration. ROI, region of interest; HOG, histogram of oriented gradient

Survival analysis was performed based on the results of spectral clustering analysis. The workflow of radiomics signature analysis is shown in Fig. 2.

### Development and evaluation of combined prognostic model

As described previously^19^, the risk factors in the NRI included age > 60 years, Eastern Cooperative Oncology Group (ECOG) score ≥ 2, elevated lactate dehydrogenase (LDH), PTI, stage II, and stage III/IV. The NRI stratified patients into low-risk (0), intermediate-low-risk (1), intermediate-high-risk (2), high-risk (3), and very high-risk (≥ 4) groups. After exclusion of stage III/IV disease, the early-stage adjusted NRI (ES-NRI) stratified into low-risk (0), intermediate-low-risk (1), intermediate-high-risk (2), and high-risk (≥ 3) groups.

A combined clinic-radiomics model, NRI plus MRI radiomics (NRI-M), was developed. The discrimination and calibration performance were evaluated by time-dependent receiver operating characteristic (tROC), corresponding area under curve (tAUC), Harrell’s C-index, calibration graph, cumulative prediction error, and integrated Brier score (IBS). Bootstrap method was used for internal verification.

### Endpoints and statistics

The primary endpoint was overall survival (OS), defined as the time from the start of treatment to death from any cause or to the last follow-up. Progression-free survival (PFS) was defined as the time from the start of treatment to disease progression, relapse, or death. Survivals were estimated by the Kaplan-Meier method and compared with log-rank tests. The univariable and multivariable analyses were used with Cox proportional hazards regression. The categorical variables between groups were compared by using χ^2^ test.

Predicted survival probabilities by applying the radiomics signature and NRI-M to the baseline survival estimate at the individual level, and averaging across each risk group. Then the predicted survival is compared with the Kaplan-Meier observed survival. The tROC, tAUC and Harrell’s C-index were used to evaluate model discrimination. The tROC and tAUC compute the sensitivity (true-positive rate) against one minus specificity (false-positive rate) for consecutive cutoffs for the predicted risk and over time. The cumulative prediction errors or IBS was used to evaluate prediction accuracy over time, with inverse of the probability of censoring weights (IPCW) to account for censoring, cross-validation used to avoid overfitting. ROI segmentation, MRI normalization, feature extraction and selection, and model construction were performed using AIMED version 1.7.5 (https://www.blothealth.com). Statistical analyses were performed using IBM SPSS Statistics, Version 24.0, and packages of “survival”, “survminer”, “timeROC”, “dynpred”, “rms”, and “pec” in R version 3.6.2 (http://www.r-project.org/).

## Results

### Patients’ characteristics and survival

The clinical characteristics are shown in Table 1. All patients had primary disease in the UADT sites. The male-to-female ratio was 2.7:1. The median age was 46 years (range, 6–85). The majority of patients originated from the nasal cavity (80.1%), presented with early-stage disease (94.3%), and had PTI (71.6%) and good performance status (ECOG score 0–1; 94.3%). With a median follow-up time of 50 months for surviving patients, the 5-year OS and PFS rates were 74.9% and 64.9% for all patients, and 76.8% and 67.9% for early-stage patients.

**Table 1:** Patient characteristics stratified by the MRI radiomics signature in patients with all-stages and early-stage ENKTCL.

			MRI radiomics signature			
	Total		Type I		Type II	
**Characteristic**	No. (%)		No. (%)		No. (%)	*P*
**All Stages (n = 176)**	176		65		111	
Gender, male	128 (72.7)		46 (70.8)		82 (73.9)	0.655
Age, > 60 years	26 (14.8)		10 (15.4)		16 (14.4)	0.861
B symptoms	82 (46.6)		22 (33.8)		60 (54.1)	0.009
ECOG score ≥ 2	10 (5.7)		1 (1.5)		9 (8.1)	0.094
Stage I–II	166 (94.3)		63 (96.9)		103 (92.8)	< 0.001
PTI	126 (71.6)		23 (35.4)		103 (92.8)	< 0.001
Elevated LDH	46 (26.1)		9 (13.8)		37 (33.3)	0.005
Primary site, nasal cavity	141 (80.1)		58 (89.2)		83 (74.8)	0.020
**Stage I–II (n = 166)**	166		63		103	
Gender, male	121 (72.9)		45 (71.4)		76 (73.8)	0.740
Age, > 60 years	25 (15.1)		10 (15.9)		15 (14.6)	0.819
B symptoms	77 (46.4)		21 (33.3)		56 (54.4)	0.008
ECOG score ≥ 2	10 (6.0)		1 (1.6)		9 (8.7)	0.091
Stage II	65 (39.2)		3 (4.8)		62 (60.2)	< 0.001
Elevated LDH	43 (25.9)		9 (14.3)		34 (33.0)	0.008
PTI	116 (69.9)		21 (33.3)		95 (92.2)	< 0.001
Primary site, nasal cavity	134 (80.7)		56 (88.9)		78 (75.7)	0.037

Abbreviations: *ENKTCL* extranodal nasal-type NK/T-cell lymphoma, *MRI* magnetic resonance imaging, *ECOG* Eastern Cooperative Oncology Group, *LDH* lactate dehydrogenase, *PTI* primary tumor invasion.

### Risk-stratified subgroups by radiomics signatures

**Fig. 3:**
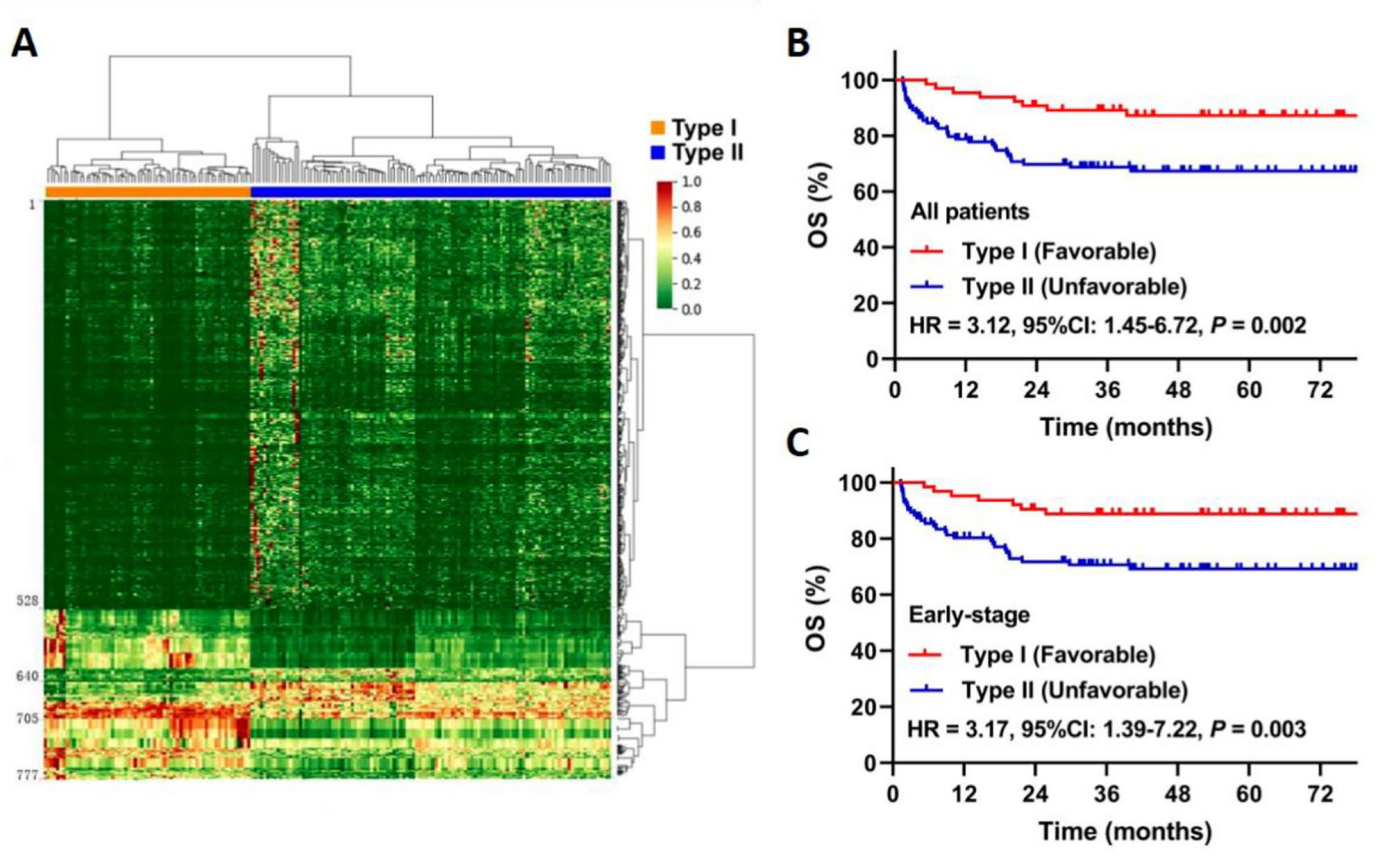
MRI-based radiomics signature and survival after classification. **A** heatmap generated by unsupervised spectral clustering of extracted radiomic features, applied to stratify patients into type I and type II; **B** the overall survival (OS) curve for all patients; **C** the OS curve for early-stage patients. HR, hazard ratio

A total of 777 features with statistical significance (*P* < 0.05) were preliminarily selected from the 3144 radiomics features in the whole group. A radiomics signature was further constructed based on the spectrum cluster analysis of unsupervised learning. The heat map of cluster analysis showed the final classification results (Fig. 3A, Supplementary Tables 1, Supplementary Table 2). In type I group, 1-528 and 641–705 are dark green, with low eigenvalues, and 529–640 and 706–777 are red, yellow, and light green, with high eigenvalues. In type II group, 1-528 and 641–705 are red, yellow, and light green, with high eigenvalues, and 529–640 and 706–777 are dark green, with low eigenvalues.

Patients with type II had significantly higher adverse prognostic factors, including B symptoms, ECOG score ≥ 2, elevated LDH, advanced-stage disease and PTI, than those with type I (Table 1). The 5-year OS was 87.2% in type I, significantly higher than 67.3% in type II (Hazard Ratio [HR] 3.12, 95% CI 1.45–6.72; *P* = 0.002; Fig. 3B). Similar results between type I and II were observed in early-stage patients (88.8% vs. 69.2%; HR 3.17, 95% CI 1.39–7.22; *P* = 0.003; Fig. 3C).

### Validation of risk-stratified groups based on MRI radiomics classifier

The AUC and Harrell’s C index of MRI radiomics classifier for predicting 5-year OS were 0.664 and 0.623 (95% CI: 0.566–0.681) for all patients, and 0.667 and 0.622 (95% CI: 0.559–0.686) for early-stage patients, respectively. The 5-year OS predicted by MRI radiomics classifier was 87.8% for type I and 66.7% for type II in the whole group, and 88.9% for type I and 69.0% for type II in the early-stage group. The predicted OS was comparable to the observed OS (Table 2). The calibration curve for the probability of 5-year OS showed good correlation between the actual observation and the radiomics signature prediction in the whole group and early-stage patients.

**Table 2:** The observed and predicted 5-year OS of the MRI-based radiomics signature and NRI-M in patients with all-stages and early-stage ENKTCL.

Risk group	No. (%)	Observed 5-year OS (%)	*P*		Predicted 5-year OS (%)
**All Stages (n = 176)**					
MRI radiomics signature			0.002		
Type I (Favorable)	65 (36.9)	87.2			87.8
Type II (Unfavorable)	111 (63.1)	67.3			66.7
NRI-M			< 0.001		
Low-risk (0–1)	53 (30.1)	90.5			90.9
Intermediate-low-risk (2)	37 (21.0)	80.8			83.5
Intermediate-high-risk (3)	47 (26.7)	69.6			70.9
High-risk (4)	28 (15.9)	63.4			52.0
Very high-risk (≥ 5)	11 (6.3)	22.7			28.8
**Stage I–II (n = 166)**					
MRI radiomics signature			0.004		
Type I (Favorable)	63 (38.0)	88.8			88.9
Type II (Unfavorable)	103 (62.0)	69.2			69.0
NRI-M			< 0.001		
Low-risk (0–1)	53 (31.9)	90.5			90.4
Intermediate-low-risk (2)	37 (22.3)	80.8			82.9
Intermediate-high-risk (3)	45 (27.1)	71.5			70.8
High-risk (≥ 4)	31 (18.7)	54.8			52.8

Abbreviations: *OS* overall survival, *MRI* magnetic resonance imaging, *NRI* nomogram-revised risk index, *NRI-M* MRI radiomics-based NRI, *ENKTCL* extranodal nasal-type NK/T-cell lymphoma

### Construction and validation of NRI-M model

**Fig. 4:**
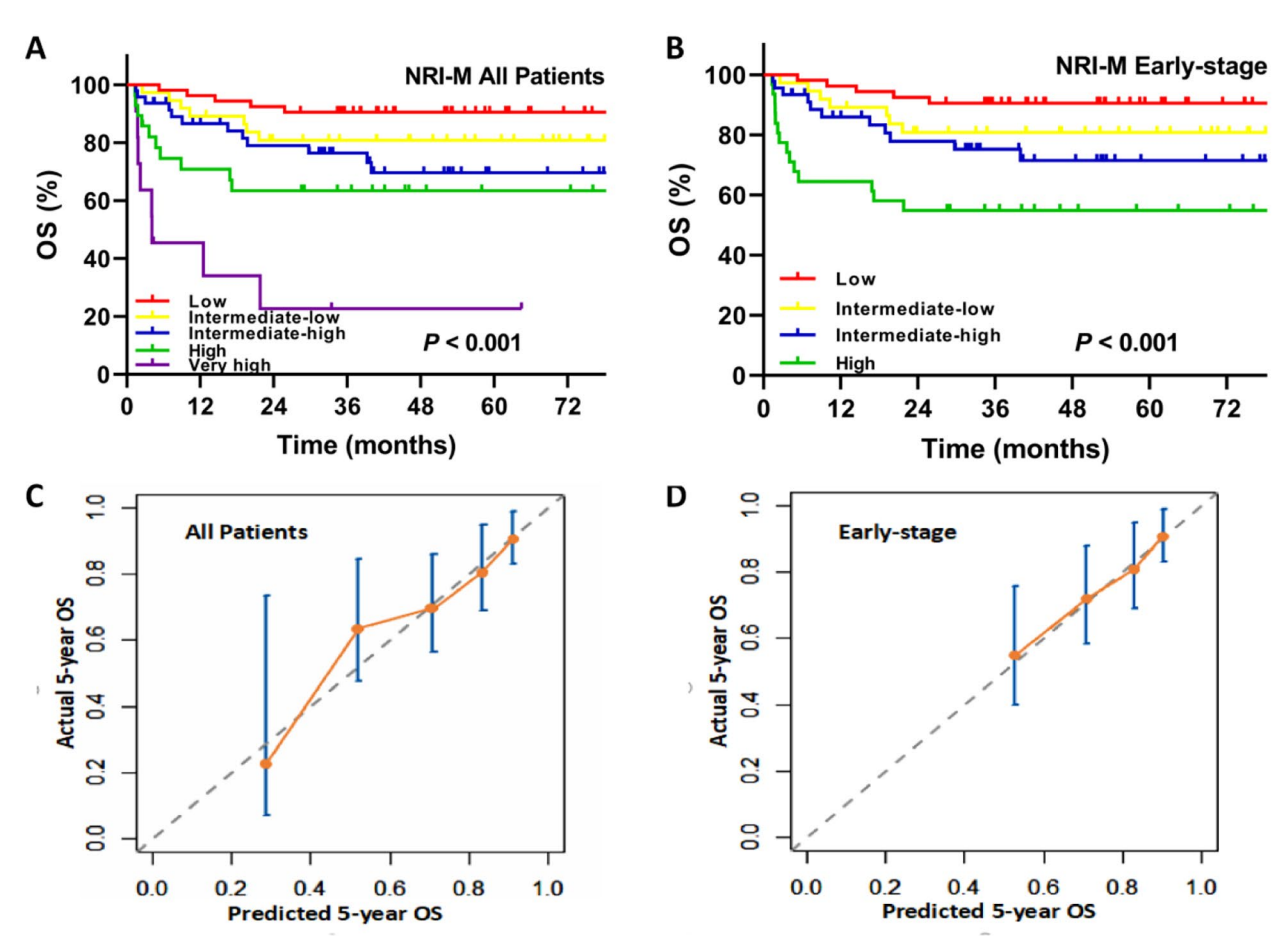
Construction and validation of the NRI-M model. The NRI-M model was developed with integration of MRI radiomics classifier into the NRI. **A** the overall survival (OS) after stratification by the NRI-M for all patients; **B** the OS after stratification by the NRI-M for early-stage patients; **C** the calibration curve of the NRI-M for all patients; **D** the calibration curve of the NRI-M for early-stage patients. MRI, magnetic resonance imaging; NRI, nomogram-revised risk index; NRI-M, NRI plus multi-modal MRI radiomics signature; AUC, area under the curve

The NRI-M model integrates MRI radiomics classifier into the NRI-defined clinical prognostic factors [19], and assigns one point-each to the type II MRI radiomics. Based on the NRI-M, the 5-year OS rates in the entire cohort were 90.5% for low-risk, 80.8% for intermediate-low-risk, 69.6% for intermediate-high-risk, 63.4% for high-risk, and 22.7% for very high-risk groups (*P* < 0.001, Fig. 4A). The corresponding OS rates in early-stage patients were 90.5% for low-risk, 80.8% for intermediate-low-risk, 71.5% for intermediate-high-risk, and 54.8% for high-risk group, respectively (*P* < 0.001, Fig. 4B).

The 5-year OS rates predicted by the NRI-M for the whole group in the low-, intermediate-low-, intermediate-high-, high-, and very high-risk groups were 90.9%, 83.5%, 70.9%, 52.0%, and 28.8%, respectively. The predicted 5-year OS for early-stage patients in the low-, intermediate-low-, intermediate-high-, and high-risk groups was 90.4%, 82.9%, 70.8%, and 52.8%, respectively (Table 2). The calibration plot for the probability of 5-year OS showed a good correlation between the actual observed outcome and the prediction by the NRI-M for all-stages (Fig. 4C) and early-stage patients (Fig. 4D).

### Evaluation of NRI-M Model

**Fig. 5:**
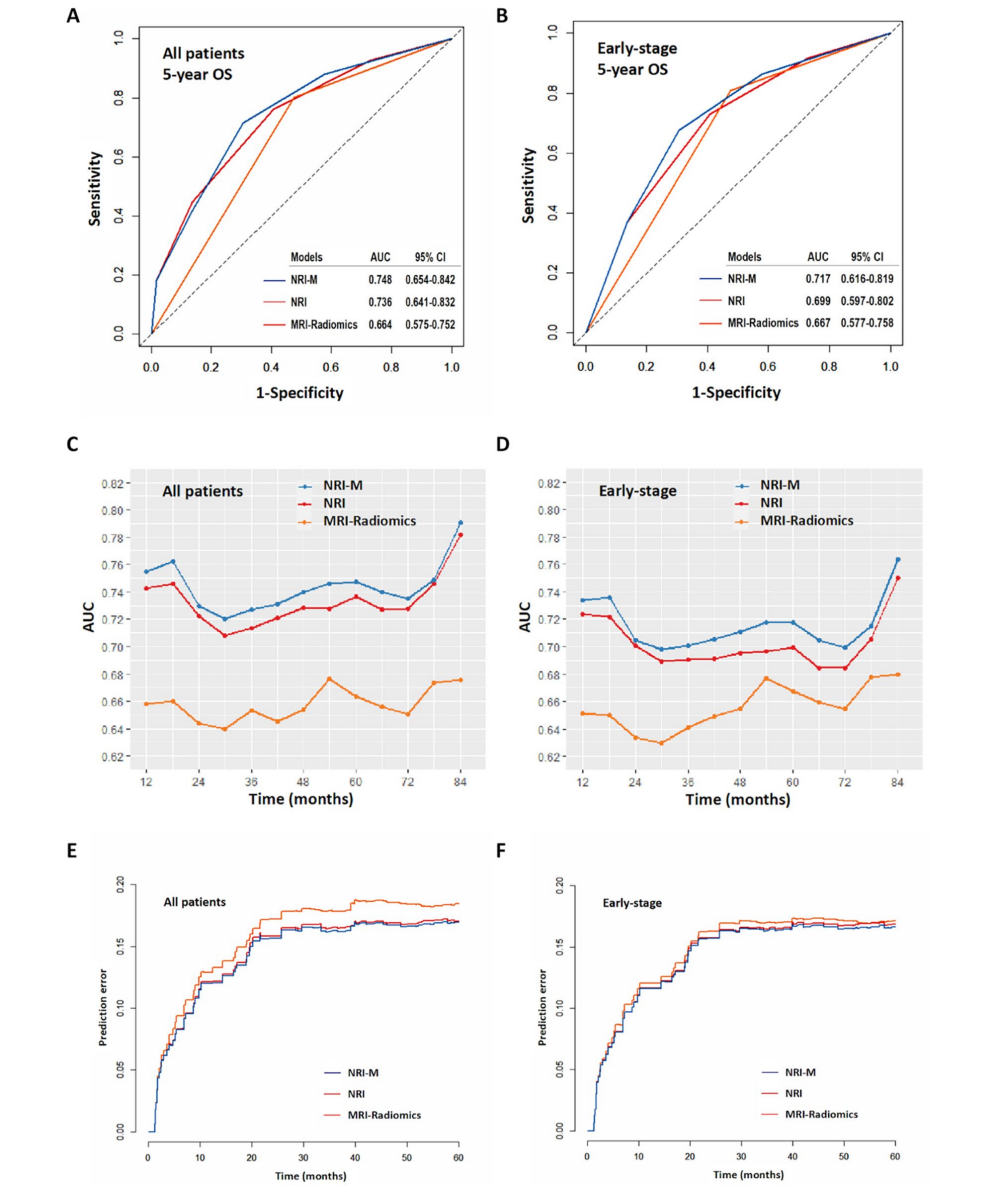
Comparison and evaluation of NRI-M model by predictive accuracy, discrimination and predictive error. **A** the ROC curve of NRI-M, NRI and MRI-Radiomics for all patients; **B** the ROC curve of NRI-M, NRI and MRI-Radiomics for early-stage patients. **C** the tAUC curve for all patients; **D** the tAUC curve for early-stage patients; **E** the prediction error curve for all patients; **F** the prediction error curve for early-stage patients. MRI, magnetic resonance imaging; NRI, nomogram-revised risk index; NRI-M, NRI plus multi-modal MRI radiomics signature; ROC, receiver operating characteristic; tAUC, time-dependent area under curve

The NRI-M model was evaluated by predictive accuracy, discrimination and predictive error. Compared with the NRI and the MRI radiomics classifier, the NRI-M model had better levels of accuracy for predicting OS. The AUC of NRI-M for predicting 5-year OS (0.748, 95%CI 0.654–0.842) for all patients was higher than that of the NRI (0.736, 95%CI 0.641–0.832) or the MRI radiomics classifier (0.664, 95%CI 0.575–0.752; *P* = 0.013, Fig. 5A). Similarly, for early-stage patients, the AUC of NRI-M (0.717, 95%CI 0.616–0.819) for predicting 5-year OS was higher than that of the NRI (0.699, 95%CI 0.597–0.802) or radiomics classifier (0.667, 95%CI 0.577–0.758; Fig. 5B). Moreover, the tAUC of the NRI-M model between 12 and 84 months was consistently higher than the MRI radiomics classifier and the NRI model in the whole group (Fig. 5C) and in the early-stage patients (Fig. 5D). Moreover, the Harrell’s C-index of the NRI-M of the whole group (0.740, 95%CI: 0.667–0.814) and early-stage patients (0.729, 95%CI: 0.649–0.810) was higher than that of the NRI (0.737, 95%CI: 0.664–0.810; 0.727, 95% CI: 0.648–0.807) and MRI radiomics classifier (0.623, 95%CI: 0.566–0.681; 0.622, 95%CI: 0.559–0.686).

The performance of NRI-M model was assessed by calculating prediction error over time in the entire and early-stage patients. In the whole group, the NRI-M IBS (0.142) of the 5-year OS was lower than that of the NRI (0.144) and MRI radiomics classifier (0.156). Similarly, in the early-stage patients, the IBS (0.140) of the NRI-M was also lower than that of the NRI (0.142) and the MRI radiomics classifier (0.146). The corresponding prediction error curves of all models were shown in the whole group (Fig. 5E) and early-stage patients (Fig. 5F). The results suggest that the NRI-M have better discrimination and accuracy for all-stages and early-stage patients.

## Discussion

Because of the disease rarity and heterogeneity of ENKTCL, any attempt to establish a clinic-biologic prognostic model is challenging. To our knowledge, this is the first study to demonstrate the prognostic effect of MRI radiomics signature in ENKTCL and validate the NRI-M model that incorporates MRI radiomics signature and NRI-defined clinical factors for risk stratification under the current treatment strategies. Patients with type II classifier tended to have more adverse clinical features and unfavorable prognosis than those with type I. The NRI-M was validated as a useful tool to predict survival and stratify ENKTCL patients. Comparison of the models’ performance showed that the NRI-M was superior to the original NRI or MRI radiomics classifier in terms of discrimination, predictive accuracy, and clinical decision guidance in the all-stages or early-stage cohort. The results suggested that NRI-M has a good prognostic and predictive ability and provides the basis for clinical trial design and clinical decision-making improvement.

Continuous optimization of risk stratification is important for the treatment decision-making and improvement of prognosis in ENKTCL [5, 17–19]. Non-invasive functional imaging such as MRI or PET, routinely used in ENKTCL, plays an important role in the diagnosis, treatment guidance, and monitoring and can further evaluate the biological characteristics of the tumor. Given traditional image interpretation is often subjective or qualitative, MRI-based radiomics analysis has become important to quantitatively investigate the association between medical imaging signature and clinical endpoints. The MRI-based radiomics classifier in this study yielded a significant association with OS and clinical features and exhibited good performance in predicting OS in both the entire and early-stage cohorts of ENKTCL patients. Compared with patients with type I, patients with type II MRI radiomics had significantly higher adverse prognostic factors such as B symptoms, advanced-stage, elevated LDH, PTI, poor performance status, and primary extra-UADT sites, clearly indicating aggressive biological behavior with a greater probability of progression or relapse.

The easy-to-use NRI model with incorporation of clinical prognostic factors was initially introduced by the China Lymphoma Collaborative Group (CLCG) to stratify patients with all-stages or early-stage ENKTCL [5, 19], and to better guide treatment decision and surveillance for early-stage disease [5, 14, 26]. Risk- and response-adapted therapy, involving radiotherapy with or without chemotherapy for low-risk groups and upfront/early radiotherapy and non-anthracycline-based chemotherapy (radiotherapy followed by chemotherapy or ≤3 cycles of brief chemotherapy followed by radiotherapy) for intermediate- and high-risk groups, is a viable and effective strategy for early-stage ENKTCL [14]. Another prognostic model with integration of blood circulating EBV-DNA estimated the survival of patients with ENKTCL in the modern chemotherapy era [18, 27]. Although several molecular biomarkers have been associated with risk of disease mortality [28–30], a clinical-radiomics model that could be used for risk-adapted treatment approaches has not been specifically addressed in ENKTCL. In this study, we further verified that the NRI-M incorporating MRI-based radiomics signature into the NRI is a powerful predictor for OS, establishing five prognostic groups. The NRI-M model showed an overall superior predictive capacity and better utility for clinical decision-making than either NRI or MRI-based radiomics classifier alone. Low-risk patients with 0–1 risk factors in the NRI-M generally have favorable prognoses with 5-year OS rate of approximately 90%. Intermediate-risk patients with 2–3 risk factors have unfavorable prognoses with the 5-year OS of 70–81%. However, high- and very high-risk patients with ≥ 4 risk factors have worse outcomes, leading to a 5-year OS of 20–60%. The NRI-M can help identify high-risk or very high-risk disease upfront and enable innovative systematic therapy. In contrast, the identification of low-risk patients with favorable prognosis is equally important as such patients could be considered for de-escalated systematic therapy in the setting of curative radiotherapy. In addition, intermediate-risk early-stage patients would benefit from more effective systematic therapy when combined with radiotherapy [11].

The strengths of this study include the normalization of MRI signal-intensity and radiomics features, unsupervised clustering, combination of clinical factors and radiomics signature, and current standard treatment. First, the data were obtained from high-quality medical imaging, and normalization was applied to build a realistic radiomics signature. To obtain objective and reliable data and eliminate useless features, MRI feature selection was performed before clustering, and then an unsupervised spectral clustering method was used to stratify patients with ENKTCL. The unsupervised clustering has provided better stability in patient stratification and prediction than conventional radiomics feature extraction techniques [24]. Second, we believe that the results of this exploratory research to build the radiomics signature and prognostic model have combined clinical factors with radiomics signature for patients with ENKTCL to address the gap in knowledge. Pretreatment risk stratification using radiomics data could provide beneficial information to physicians that would enable them to deliver treatment tailored to each patient’s individual risk, without further radiation exposure. Moreover, the patients primarily treated with current standard treatment strategies improved the reliability of the conclusions.

The study limitations include the lack of external validation, other biological data and genomic variables, the analysis of different delineation algorithms, and the comparison of feature selection algorithms. First, we only conducted internal verification. Expansion of recent data of our institution or external institution for further analysis could evaluate the NRI-M model more accurately. Second, genomic or molecular biomarker information such as EBV-DNA is not included [30]. However, the NRI-M has shown the advantages of combining clinical factors with omics, providing a basis for integrating a more comprehensive clinicopathology and multi-omics risk model in the future [28–30]. Third, manual segmentations were performed in this study. Compared with semi-automatic segmentation, it is time-consuming and not always feasible on large datasets. Manual segmentation by different people does have certain subjectivity. However, as reported in a study [31]: “Radiologists can flexibly inhibit targets manually resulting in highly accurate segmentations”. Further comparisons can be made by comparing manual and semi-automatic segmentation to see how much change will occur in the final results. Finally, the well-established feature selection algorithms, such as the LASSO and hybrid methods [32, 33], were not compared in this study. The comparison of these algorithms will show a number of interesting and useful results and suggests promising directions for future research in this area. The matRadiomics, a novel and complete radiomics framework, may simplify and optimize the whole comparison process [34].

## Conclusions

In summary, the NRI-M model shows better prognostic discrimination and stratification than either MRI radiomics signature or NRI alone. It facilitates the establishment of a more comprehensive clinic-biologic risk model and the design of prospective clinical studies in patients with ENKTCL.

## Electronic supplementary material

Below is the link to the electronic supplementary material.

Supplementary Material 1

Supplementary Material 2

## Data Availability

The datasets generated and analysed during the current study are not publicly available, but are available from the corresponding author on reasonable request.

## Abbreviations

CI: Confidence interval
CLCG: China Lymphoma Collaborative Group
DCEI: Dynamic contrast-enhanced T1-weighted imaging
DWI: Diffusion-weighted imaging
EBV: Epstein-Barr virus
ECOG: Eastern Cooperative Oncology Group
HOG: Histogram of oriented gradient
HR: Hazard ratio
IBS: Integrated Brier score
LDH: Lactate dehydrogenase
ENKTCL: Extranodal nasal-type NK/T-cell lymphoma
MC: Marching Cube
NRI: Nomogram-revised risk index
NRI-M: Nomogram-revised risk index plus MRI radiomics
OS: Overall survival
PFS: Progression-free survival
PTI: Primary tumor invasion
tAUC: Time-dependent area under curve
TE: Echo time
TR: Repetition time
tROC: Time-dependent receiver operating characteristic
T1WI: T1 weighted imaging
T2WI: T2 weighted imaging
UADT: Upper aerodigestive tract
