# Supplementary material for: Risk stratification and prognostic value of multi-modal MRI-based radiomics for extranodal nasal-type NK/T-cell lymphoma
Source: BMC Cancer. 2023 Jan 25;23:88. doi: 10.1186/s12885-023-10557-3 (PMC9878926; doi:10.1186/s12885-023-10557-3)
Supplement: Supplementary file 1 — Supplementary Material 1 [file 12885_2023_10557_MOESM1_ESM.docx]

| No. | Feature name | No. | Feature name |
| --- | --- | --- | --- |
| 1 | Texture-GLZSM-Large Zone / High Gray Emphasis | 40 | Hog-0_1_0_3 |
| 2 | Texture-GLZSM-Gray-Level Non-Uniformity | 41 | Hog-1_1_0_3 |
| 3 | Texture-GLZSM-Zone Size Non-Uniformity | 42 | Hog-2_1_0_3 |
| 4 | Wavelet-LLL - Histogram - Mean | 43 | Hog-2_2_0_3 |
| 5 | Wavelet-LLL - Histogram - Energy | 44 | Hog-0_1_1_3 |
| 6 | Wavelet-LLL - Histogram - Entropy | 45 | Hog-1_0_2_3 |
| 7 | Wavelet-LLH - Histogram - Variance | 46 | Hog-0_0_0_4 |
| 8 | Wavelet-LLH - Histogram - Kurtosis | 47 | Hog-1_2_0_4 |
| 9 | Wavelet-LLH - Histogram - Energy | 48 | Hog-0_0_1_4 |
| 10 | Wavelet-LLH - Histogram - Entropy | 49 | Hog-2_0_0_5 |
| 11 | Wavelet-LHL - Histogram - Variance | 50 | Hog-0_1_0_5 |
| 12 | Wavelet-LHL - Histogram - Skewness | 51 | Hog-1_0_2_5 |
| 13 | Wavelet-LHL - Histogram - Entropy | 52 | Hog-0_0_0_6 |
| 14 | Wavelet-LHH - Histogram - Skewness | 53 | Hog-0_0_1_6 |
| 15 | Hog-2_0_0_0 | 54 | Hog-0_0_2_6 |
| 16 | Hog-0_1_0_0 | 55 | Hog-2_1_0_7 |
| 17 | Hog-1_1_0_0 | 56 | Hog-0_2_0_7 |
| 18 | Hog-2_1_0_0 | 57 | Hog-2_2_0_7 |
| 19 | Hog-0_2_0_0 | 58 | Hog-0_1_1_7 |
| 20 | Hog-0_0_1_0 | 59 | Hog-2_1_1_7 |
| 21 | Hog-2_0_1_0 | 60 | Hog-0_2_1_7 |
| 22 | Hog-0_1_1_0 | 61 | Hog-2_1_2_7 |
| 23 | Hog-2_1_1_0 | 62 | Hog-0_2_2_7 |
| 24 | Hog-1_2_1_0 | 63 | Hog-2_0_0_8 |
| 25 | Hog-2_0_2_0 | 64 | Hog-0_1_0_8 |
| 26 | Hog-0_0_0_1 | 65 | Hog-1_1_0_8 |
| 27 | Hog-1_0_0_1 | 66 | Hog-2_1_0_8 |
| 28 | Hog-2_0_0_1 | 67 | Hog-0_2_0_8 |
| 29 | Hog-0_1_0_1 | 68 | Hog-1_2_0_8 |
| 30 | Hog-1_1_0_1 | 69 | Hog-2_2_0_8 |
| 31 | Hog-0_1_1_1 | 70 | Hog-2_0_1_8 |
| 32 | Hog-1_1_2_1 | 71 | Hog-2_1_1_8 |
| 33 | Hog-0_0_0_2 | 72 | Hog-1_2_1_8 |
| 34 | Hog-1_1_0_2 | 73 | Hog-1_0_2_8 |
| 35 | Hog-2_2_0_2 | 74 | Hog-0_0_0_9 |
| 36 | Hog-0_0_1_2 | 75 | Hog-2_0_0_9 |
| 37 | Hog-1_0_1_2 | 76 | Hog-0_1_0_9 |
| 38 | Hog-0_2_1_2 | 77 | Hog-1_1_0_9 |
| 39 | Hog-1_0_0_3 | 78 | Hog-2_1_0_9 |

**Supplementary Table 1.** The features of the multi-modal MRI radiomics signature.

| No. | Feature name | No. | Feature name |
| --- | --- | --- | --- |
| 79 | Hog-2_2_0_9 | 118 | Hog-2_0_0_14 |
| 80 | Hog-2_0_1_9 | 119 | Hog-0_0_2_14 |
| 81 | Hog-1_1_1_9 | 120 | Hog-0_1_2_14 |
| 82 | Hog-2_1_1_9 | 121 | Hog-0_2_2_14 |
| 83 | Hog-0_2_1_9 | 122 | Hog-2_0_0_15 |
| 84 | Hog-0_0_2_9 | 123 | Hog-0_0_2_15 |
| 85 | Hog-1_0_0_10 | 124 | Hog-0_1_2_15 |
| 86 | Hog-2_0_0_10 | 125 | Hog-1_0_0_16 |
| 87 | Hog-2_1_0_10 | 126 | Hog-2_1_1_16 |
| 88 | Hog-1_0_1_10 | 127 | Hog-2_0_2_16 |
| 89 | Hog-2_0_1_10 | 128 | Hog-0_0_0_17 |
| 90 | Hog-2_1_1_10 | 129 | Hog-2_1_0_17 |
| 91 | Hog-1_0_2_10 | 130 | Hog-2_2_0_17 |
| 92 | Hog-2_0_2_10 | 131 | Hog-0_0_1_17 |
| 93 | Hog-0_2_2_10 | 132 | Hog-2_1_1_17 |
| 94 | Hog-0_0_0_11 | 133 | Hog-0_0_2_17 |
| 95 | Hog-1_0_0_11 | 134 | Hog-2_1_2_17 |
| 96 | Hog-2_0_0_11 | 135 | Hog-2_0_0_18 |
| 97 | Hog-1_1_0_11 | 136 | Hog-0_1_0_18 |
| 98 | Hog-2_1_0_11 | 137 | Hog-1_1_0_18 |
| 99 | Hog-0_0_1_11 | 138 | Hog-2_1_0_18 |
| 100 | Hog-1_0_1_11 | 139 | Hog-2_2_0_18 |
| 101 | Hog-2_0_1_11 | 140 | Hog-2_0_1_18 |
| 102 | Hog-0_1_1_11 | 141 | Hog-1_1_1_18 |
| 103 | Hog-1_1_1_11 | 142 | Hog-2_2_1_18 |
| 104 | Hog-0_0_2_11 | 143 | Hog-1_0_2_18 |
| 105 | Hog-1_0_2_11 | 144 | Hog-0_0_0_19 |
| 106 | Hog-2_1_0_12 | 145 | Hog-0_1_0_19 |
| 107 | Hog-2_2_0_12 | 146 | Hog-1_1_0_19 |
| 108 | Hog-0_2_1_12 | 147 | Hog-2_1_0_19 |
| 109 | Hog-2_1_2_12 | 148 | Hog-0_2_0_19 |
| 110 | Hog-0_2_2_12 | 149 | Hog-0_0_1_19 |
| 111 | Hog-0_0_0_13 | 150 | Hog-1_0_1_19 |
| 112 | Hog-1_1_0_13 | 151 | Hog-2_0_1_19 |
| 113 | Hog-2_1_0_13 | 152 | Hog-2_1_1_19 |
| 114 | Hog-1_2_0_13 | 153 | Hog-0_2_1_19 |
| 115 | Hog-2_2_0_13 | 154 | Statistics-[0, 1, 0]-Contrast |
| 116 | Hog-0_2_1_13 | 155 | Statistics-[0, 1, 0]-Inertia |
| 117 | Hog-2_0_2_13 | 156 | Statistics-[0, 1, 0]-Inverse Variance |

**Supplementary Table 1 (continued)**

| No. | Feature name | No. | Feature name |
| --- | --- | --- | --- |
| 157 | Statistics-[-1, 1, 0]-Contrast | 189 | Texture-GLZSM-Large Zone / High Gray Emphasis |
| 158 | Statistics-[-1, 1, 0]-Inertia | 190 | Texture-GLZSM-Gray-Level Non-Uniformity |
| 159 | Statistics-[-1, -1, 0]-Contrast | 191 | Texture-GLZSM-Zone Size Non-Uniformity |
| 160 | Statistics-[-1, -1, 0]-Inertia | 192 | Texture-GLZSM-Zone Size Percentage |
| 161 | Statistics-[0, 1, -1]-Contrast | 193 | Wavelet-LLL - Histogram - Variance |
| 162 | Statistics-[0, 1, -1]-Inertia | 194 | Wavelet-LLL - Histogram - Kurtosis |
| 163 | Statistics-[0, 1, -1]-Inverse Variance | 195 | Wavelet-LLL - Histogram - Energy |
| 164 | Statistics-[0, 0, -1]-Contrast | 196 | Wavelet-LLL - Histogram - Entropy |
| 165 | Statistics-[0, 0, -1]-Inertia | 197 | Wavelet-LLH - Histogram - Variance |
| 166 | Statistics-[0, 0, -1]-Inverse Variance | 198 | Wavelet-LHL - Histogram - Variance |
| 167 | Statistics-[0, -1, -1]-Contrast | 199 | Wavelet-LHL - Histogram - Skewness |
| 168 | Statistics-[0, -1, -1]-Inertia | 200 | Wavelet-LHL - Histogram - Kurtosis |
| 169 | Statistics-[0, -1, -1]-Inverse Variance | 201 | Wavelet-LHL - Histogram - Energy |
| 170 | Statistics-[-1, 1, -1]-Contrast | 202 | Wavelet-LHL - Histogram - Entropy |
| 171 | Statistics-[-1, 1, -1]-Inertia | 203 | Wavelet-LHH - Histogram - Variance |
| 172 | Statistics-[1, -1, -1]-Contrast | 204 | Wavelet-HLL - Histogram - Variance |
| 173 | Statistics-[1, -1, -1]-Inertia | 205 | Wavelet-HLL - Histogram - Energy |
| 174 | Statistics-[-1, -1, -1]-Contrast | 206 | Wavelet-HLL - Histogram - Entropy |
| 175 | Statistics-[-1, -1, -1]-Inertia | 207 | Wavelet-HLH - Histogram - Variance |
| 176 | Statistics-[1, 1, -1]-Contrast | 208 | Wavelet-HHL - Histogram - Variance |
| 177 | Statistics-[1, 1, -1]-Inertia | 209 | Wavelet-HHL - Histogram - Kurtosis |
| 178 | Texture-Histogram-Variance | 210 | Wavelet-HHL - Histogram - Energy |
| 179 | Texture-Histogram-Skewness | 211 | Wavelet-HHL - Histogram - Entropy |
| 180 | Texture-Histogram-Kurtosis | 212 | Wavelet-HHH - Histogram - Variance |
| 181 | Texture-Histogram-Energy | 213 | Hog-1_0_0_0 |
| 182 | Texture-Histogram-Entropy | 214 | Hog-2_0_0_0 |
| 183 | Texture-GTSDM-Sum Entropy | 215 | Hog-2_1_0_0 |
| 184 | Texture-GTSDM-Entropy | 216 | Hog-0_2_0_0 |
| 185 | Texture-GTSDM-Difference Entropy | 217 | Hog-0_2_1_0 |
| 186 | Texture-GTSDM-Information Correlation 2 | 218 | Hog-2_0_2_0 |
| 187 | Texture-GLZSM-Small Zone Size Emphasis | 219 | Hog-0_0_0_1 |
| 188 | Texture-GLZSM-Small Zone / Low Gray Emphasis | 220 | Hog-2_0_0_1 |

**Supplementary Table 1 (continued)**

| No. | Feature name | No. | Feature name |
| --- | --- | --- | --- |
| 221 | Hog-0_1_0_1 | 261 | Hog-2_1_1_9 |
| 222 | Hog-2_1_2_1 | 262 | Hog-1_2_1_9 |
| 223 | Hog-0_0_0_2 | 263 | Hog-2_0_2_9 |
| 224 | Hog-2_0_0_2 | 264 | Hog-1_1_2_9 |
| 225 | Hog-1_1_0_2 | 265 | Hog-1_2_2_9 |
| 226 | Hog-0_2_0_2 | 266 | Hog-0_0_0_10 |
| 227 | Hog-2_2_0_2 | 267 | Hog-1_0_0_10 |
| 228 | Hog-2_1_0_3 | 268 | Hog-2_0_0_10 |
| 229 | Hog-1_2_0_3 | 269 | Hog-0_1_1_10 |
| 230 | Hog-2_2_0_3 | 270 | Hog-0_0_2_10 |
| 231 | Hog-2_1_2_3 | 271 | Hog-1_0_2_10 |
| 232 | Hog-2_2_2_3 | 272 | Hog-2_0_2_10 |
| 233 | Hog-0_0_0_4 | 273 | Hog-1_0_0_11 |
| 234 | Hog-2_0_0_4 | 274 | Hog-1_0_1_11 |
| 235 | Hog-0_2_0_4 | 275 | Hog-2_0_1_11 |
| 236 | Hog-2_2_2_4 | 276 | Hog-2_1_1_11 |
| 237 | Hog-0_1_0_5 | 277 | Hog-2_0_2_11 |
| 238 | Hog-2_0_1_5 | 278 | Hog-0_2_2_11 |
| 239 | Hog-0_0_2_5 | 279 | Hog-1_2_0_12 |
| 240 | Hog-0_0_0_6 | 280 | Hog-2_2_0_12 |
| 241 | Hog-2_1_0_6 | 281 | Hog-0_0_2_12 |
| 242 | Hog-0_0_0_7 | 282 | Hog-2_1_2_12 |
| 243 | Hog-2_2_0_7 | 283 | Hog-0_2_2_12 |
| 244 | Hog-0_0_1_7 | 284 | Hog-2_0_0_13 |
| 245 | Hog-0_2_2_7 | 285 | Hog-0_1_0_13 |
| 246 | Hog-1_0_0_8 | 286 | Hog-1_1_0_13 |
| 247 | Hog-2_0_0_8 | 287 | Hog-2_2_0_13 |
| 248 | Hog-0_1_0_8 | 288 | Hog-1_0_1_13 |
| 249 | Hog-2_1_0_8 | 289 | Hog-2_0_2_13 |
| 250 | Hog-0_2_0_8 | 290 | Hog-0_1_2_13 |
| 251 | Hog-1_0_1_8 | 291 | Hog-2_2_2_13 |
| 252 | Hog-1_1_1_8 | 292 | Hog-2_0_1_14 |
| 253 | Hog-2_0_2_8 | 293 | Hog-0_0_2_14 |
| 254 | Hog-1_1_2_8 | 294 | Hog-2_1_2_14 |
| 255 | Hog-2_1_2_8 | 295 | Hog-0_2_2_14 |
| 256 | Hog-1_0_0_9 | 296 | Hog-2_1_0_15 |
| 257 | Hog-1_1_0_9 | 297 | Hog-0_0_2_15 |
| 258 | Hog-0_2_0_9 | 298 | Hog-0_1_2_15 |
| 259 | Hog-1_0_1_9 | 299 | Hog-0_0_0_16 |
| 260 | Hog-1_1_1_9 | 300 | Hog-2_0_0_16 |

**Supplementary Table 1 (continued)**

**Supplementary Table 1 (continued)**

| No. | Feature name | No. | Feature name |
| --- | --- | --- | --- |
| 301 | Hog-2_0_1_16 | 337 | Statistics-[-1, -1, 0]-Variance |
| 302 | Hog-0_0_2_16 | 338 | Statistics-[-1, -1, 0]-Inertia |
| 303 | Hog-2_2_0_17 | 339 | Statistics-[-1, -1, 0]-Cluster Shade |
| 304 | Hog-2_0_1_17 | 340 | Statistics-[-1, -1, 0]-Cluster Tendency |
| 305 | Hog-1_1_0_18 | 341 | Statistics-[0, 1, -1]-Entropy |
| 306 | Hog-1_0_1_18 | 342 | Statistics-[0, 1, -1]-Contrast |
| 307 | Hog-0_1_1_18 | 343 | Statistics-[0, 1, -1]-Variance |
| 308 | Hog-0_0_2_18 | 344 | Statistics-[0, 1, -1]-Inertia |
| 309 | Hog-1_0_2_18 | 345 | Statistics-[0, 1, -1]-Cluster Shade |
| 310 | Hog-1_1_0_19 | 346 | Statistics-[0, 1, -1]-Cluster Tendency |
| 311 | Hog-0_2_0_19 | 347 | Statistics-[0, 1, -1]-Inverse Variance |
| 312 | Hog-2_2_0_19 | 348 | Statistics-[0, 0, -1]-Entropy |
| 313 | Hog-1_0_1_19 | 349 | Statistics-[0, 0, -1]-Contrast |
| 314 | Hog-1_1_1_19 | 350 | Statistics-[0, 0, -1]-Variance |
| 315 | Hog-2_1_1_19 | 351 | Statistics-[0, 0, -1]-Inertia |
| 316 | Hog-0_0_2_19 | 352 | Statistics-[0, 0, -1]-Cluster Shade |
| 317 | Hog-1_0_2_19 | 353 | Statistics-[0, 0, -1]-Cluster Tendency |
| 318 | Hog-2_0_2_19 | 354 | Statistics-[0, 0, -1]-Inverse Variance |
| 319 | Hog-1_1_2_19 | 355 | Statistics-[0, -1, -1]-Entropy |
| 320 | Hog-1_2_2_19 | 356 | Statistics-[0, -1, -1]-Contrast |
| 321 | Statistics-[0, 1, 0]-Entropy | 357 | Statistics-[0, -1, -1]-Variance |
| 322 | Statistics-[0, 1, 0]-Contrast | 358 | Statistics-[0, -1, -1]-Inertia |
| 323 | Statistics-[0, 1, 0]-Variance | 359 | Statistics-[0, -1, -1]-Cluster Shade |
| 324 | Statistics-[0, 1, 0]-Inertia | 360 | Statistics-[0, -1, -1]-Cluster Tendency |
| 325 | Statistics-[0, 1, 0]-Cluster Shade | 361 | Statistics-[0, -1, -1]-Inverse Variance |
| 326 | Statistics-[0, 1, 0]-Cluster Tendency | 362 | Statistics-[-1, 0, -1]-Variance |
| 327 | Statistics-[0, 1, 0]-Inverse Variance | 363 | Statistics-[-1, 0, -1]-Cluster Shade |
| 328 | Statistics-[-1, 1, 0]-Contrast | 364 | Statistics-[-1, 0, -1]-Cluster Tendency |
| 329 | Statistics-[-1, 1, 0]-Variance | 365 | Statistics-[1, 0, -1]-Variance |
| 330 | Statistics-[-1, 1, 0]-Inertia | 366 | Statistics-[1, 0, -1]-Cluster Shade |
| 331 | Statistics-[-1, 1, 0]-Cluster Shade | 367 | Statistics-[1, 0, -1]-Cluster Tendency |
| 332 | Statistics-[-1, 1, 0]-Cluster Tendency | 368 | Statistics-[-1, 1, -1]-Contrast |
| 333 | Statistics-[-1, 0, 0]-Variance | 369 | Statistics-[-1, 1, -1]-Variance |
| 334 | Statistics-[-1, 0, 0]-Cluster Shade | 370 | Statistics-[-1, 1, -1]-Inertia |
| 335 | Statistics-[-1, 0, 0]-Cluster Tendency | 371 | Statistics-[-1, 1, -1]-Cluster Shade |
| 336 | Statistics-[-1, -1, 0]-Contrast | 372 | Statistics-[-1, 1, -1]-Cluster Tendency |

| No. | Feature name | No. | Feature name |
| --- | --- | --- | --- |
| 373 | Statistics-[1, -1, -1]-Contrast | 406 | Wavelet-LHL - Histogram - Variance |
| 374 | Statistics-[1, -1, -1]-Variance | 407 | Wavelet-LHL - Histogram - Energy |
| 375 | Statistics-[1, -1, -1]-Inertia | 408 | Wavelet-LHL - Histogram - Entropy |
| 376 | Statistics-[1, -1, -1]-Cluster Shade | 409 | Wavelet-LHH - Histogram - Variance |
| 377 | Statistics-[1, -1, -1]-Cluster Tendency | 410 | Wavelet-LHH - Histogram - Kurtosis |
| 378 | Statistics-[-1, -1, -1]-Contrast | 411 | Wavelet-HLL - Histogram - Variance |
| 379 | Statistics-[-1, -1, -1]-Variance | 412 | Wavelet-HLH - Histogram - Variance |
| 380 | Statistics-[-1, -1, -1]-Inertia | 413 | Wavelet-HHL - Histogram - Mean |
| 381 | Statistics-[-1, -1, -1]-Cluster Shade | 414 | Wavelet-HHL - Histogram - Skewness |
| 382 | Statistics-[-1, -1, -1]-Cluster Tendency | 415 | Wavelet-HHL - Histogram - Energy |
| 383 | Statistics-[-1, -1, -1]-Inverse Variance | 416 | Hog-1_0_0_0 |
| 384 | Statistics-[1, 1, -1]-Contrast | 417 | Hog-2_0_0_0 |
| 385 | Statistics-[1, 1, -1]-Variance | 418 | Hog-0_1_0_0 |
| 386 | Statistics-[1, 1, -1]-Inertia | 419 | Hog-2_1_0_0 |
| 387 | Statistics-[1, 1, -1]-Cluster Shade | 420 | Hog-0_2_0_0 |
| 388 | Statistics-[1, 1, -1]-Cluster Tendency | 421 | Hog-1_2_0_0 |
| 389 | Statistics-[1, 1, -1]-Inverse Variance | 422 | Hog-0_0_1_0 |
| 390 | Texture-Histogram-Skewness | 423 | Hog-1_1_1_0 |
| 391 | Texture-Histogram-Kurtosis | 424 | Hog-2_0_2_0 |
| 392 | Texture-Histogram-Energy | 425 | Hog-1_1_2_0 |
| 393 | Texture-Histogram-Entropy | 426 | Hog-2_1_2_0 |
| 394 | Texture-GTSDM-Sum Entropy | 427 | Hog-0_2_2_0 |
| 395 | Texture-GTSDM-Entropy | 428 | Hog-1_2_2_0 |
| 396 | Texture-GTSDM-Difference Entropy | 429 | Hog-1_0_0_1 |
| 397 | Texture-GLZSM-Small Zone Size Emphasis | 430 | Hog-0_1_0_1 |
| 398 | Texture-GLZSM-Small Zone / Low Gray Emphasis | 431 | Hog-0_2_0_1 |
| 399 | Texture-GLZSM-Large Zone / High Gray Emphasis | 432 | Hog-0_0_2_1 |
| 400 | Texture-GLZSM-Gray-Level Non-Uniformity | 433 | Hog-1_0_2_1 |
| 401 | Texture-GLZSM-Zone Size Non-Uniformity | 434 | Hog-2_0_2_1 |
| 402 | Texture-GLZSM-Zone Size Percentage | 435 | Hog-2_1_2_1 |
| 403 | Wavelet-LLL - Histogram - Energy | 436 | Hog-2_2_2_1 |
| 404 | Wavelet-LLH - Histogram - Variance | 437 | Hog-1_0_0_2 |
| 405 | Wavelet-LLH - Histogram - Entropy | 438 | Hog-1_1_0_2 |

**Supplementary Table 1 (continued)**

| No. | Feature name | No. | Feature name |
| --- | --- | --- | --- |
| 439 | Hog-0_2_0_2 | 518 | Hog-1_2_0_10 |
| 440 | Hog-1_2_0_2 | 519 | Hog-0_0_1_10 |
| 481 | Hog-0_2_2_7 | 520 | Hog-1_1_1_10 |
| 482 | Hog-2_2_2_7 | 521 | Hog-2_2_1_10 |
| 483 | Hog-1_0_0_8 | 522 | Hog-0_0_2_10 |
| 484 | Hog-0_1_0_8 | 523 | Hog-2_1_2_10 |
| 485 | Hog-2_1_0_8 | 524 | Hog-1_2_2_10 |
| 486 | Hog-0_2_0_8 | 525 | Hog-1_0_0_11 |
| 487 | Hog-1_2_0_8 | 526 | Hog-0_1_0_11 |
| 488 | Hog-0_0_1_8 | 527 | Hog-2_1_0_11 |
| 489 | Hog-1_0_1_8 | 528 | Hog-1_0_1_11 |
| 490 | Hog-0_1_1_8 | 529 | Hog-0_1_1_11 |
| 491 | Hog-1_1_1_8 | 530 | Hog-2_1_1_11 |
| 492 | Hog-2_1_1_8 | 531 | Hog-0_0_2_11 |
| 493 | Hog-2_2_1_8 | 532 | Hog-2_0_2_11 |
| 494 | Hog-1_0_2_8 | 533 | Hog-0_1_2_11 |
| 495 | Hog-1_1_2_8 | 534 | Hog-2_1_2_11 |
| 496 | Hog-2_1_2_8 | 535 | Hog-0_2_2_11 |
| 497 | Hog-0_2_2_8 | 536 | Hog-2_2_2_11 |
| 498 | Hog-1_2_2_8 | 537 | Hog-0_1_0_12 |
| 499 | Hog-2_2_2_8 | 538 | Hog-1_1_0_12 |
| 500 | Hog-1_0_0_9 | 539 | Hog-2_1_0_12 |
| 501 | Hog-0_1_0_9 | 540 | Hog-0_2_0_12 |
| 502 | Hog-1_1_0_9 | 541 | Hog-1_2_0_12 |
| 503 | Hog-2_1_0_9 | 542 | Hog-2_2_0_12 |
| 504 | Hog-1_2_0_9 | 543 | Hog-0_1_1_12 |
| 505 | Hog-0_1_1_9 | 544 | Hog-0_0_2_12 |
| 506 | Hog-1_1_1_9 | 545 | Hog-1_0_2_12 |
| 507 | Hog-2_1_1_9 | 546 | Hog-0_1_2_12 |
| 508 | Hog-1_0_2_9 | 547 | Hog-0_0_0_13 |
| 509 | Hog-2_0_2_9 | 548 | Hog-1_1_0_13 |
| 510 | Hog-0_1_2_9 | 549 | Hog-1_0_1_13 |
| 511 | Hog-2_1_2_9 | 550 | Hog-2_0_1_13 |
| 512 | Hog-1_2_2_9 | 551 | Hog-1_2_1_13 |
| 513 | Hog-0_0_0_10 | 552 | Hog-0_1_2_13 |
| 514 | Hog-1_0_0_10 | 553 | Hog-1_1_2_13 |
| 515 | Hog-2_0_0_10 | 554 | Hog-2_2_2_13 |
| 516 | Hog-1_1_0_10 | 555 | Hog-0_1_0_14 |
| 517 | Hog-2_1_0_10 | 556 | Hog-1_1_0_14 |

**Supplementary Table 1 (continued)**

| No. | Feature name | No. | Feature name |
| --- | --- | --- | --- |
| 557 | Hog-2_1_0_14 | 632 | Wavelet-HHL - Histogram - Energy |
| 558 | Hog-2_2_0_14 | 633 | Wavelet-HHL - Histogram - Entropy |
| 559 | Hog-0_0_2_14 | 634 | Wavelet-HHH - Histogram - Variance |
| 560 | Hog-0_1_2_14 | 635 | Hog-2_0_0_0 |
| 601 | Hog-1_2_1_19 | 636 | Hog-0_2_0_0 |
| 602 | Hog-2_2_1_19 | 637 | Hog-2_0_1_0 |
| 603 | Hog-1_0_2_19 | 638 | Hog-2_1_1_0 |
| 604 | Hog-1_1_2_19 | 639 | Hog-1_2_2_0 |
| 605 | Hog-2_1_2_19 | 640 | Hog-2_0_0_1 |
| 606 | Statistics-[0, 1, 0]-Inverse Variance | 641 | Hog-2_1_0_1 |
| 607 | Statistics-[0, 1, -1]-Inverse Variance | 642 | Hog-2_0_1_1 |
| 608 | Statistics-[0, 0, -1]-Inverse Variance | 643 | Hog-2_1_1_1 |
| 609 | Statistics-[0, -1, -1]-Inverse Variance | 644 | Hog-1_2_0_2 |
| 610 | Statistics-[-1, -1, -1]-Inverse Variance | 645 | Hog-1_2_2_2 |
| 611 | Texture-Histogram-Variance | 646 | Hog-1_1_0_3 |
| 612 | Texture-Histogram-Energy | 647 | Hog-2_1_0_3 |
| 613 | Texture-Histogram-Entropy | 648 | Hog-2_1_2_3 |
| 614 | Texture-GTSDM-Sum Entropy | 649 | Hog-2_2_2_3 |
| 615 | Texture-GTSDM-Difference Entropy | 650 | Hog-2_0_0_4 |
| 616 | Texture-GLZSM-Small Zone Size Emphasis | 651 | Hog-2_1_0_4 |
| 617 | Texture-GLZSM-Gray-Level Non-Uniformity | 652 | Hog-1_2_2_4 |
| 618 | Texture-GLZSM-Zone Size Non-Uniformity | 653 | Hog-0_0_0_5 |
| 619 | Texture-GLZSM-Zone Size Percentage | 654 | Hog-2_0_0_5 |
| 620 | Wavelet-LLL - Histogram - Variance | 655 | Hog-0_0_0_6 |
| 621 | Wavelet-LLH - Histogram - Kurtosis | 656 | Hog-2_0_0_6 |
| 622 | Wavelet-LHL - Histogram - Variance | 657 | Hog-0_0_1_6 |
| 623 | Wavelet-LHL - Histogram - Energy | 658 | Hog-2_0_1_6 |
| 624 | Wavelet-LHL - Histogram - Entropy | 659 | Hog-0_2_0_7 |
| 625 | Wavelet-LHH - Histogram - Variance | 660 | Hog-0_2_1_7 |
| 626 | Wavelet-HLL - Histogram - Variance | 661 | Hog-2_1_2_7 |
| 627 | Wavelet-HLL - Histogram - Energy | 662 | Hog-0_1_0_8 |
| 628 | Wavelet-HLL - Histogram - Entropy | 663 | Hog-2_1_0_8 |
| 629 | Wavelet-HLH - Histogram - Variance | 664 | Hog-0_2_0_8 |
| 630 | Wavelet-HLH - Histogram - Entropy | 665 | Hog-1_2_0_8 |
| 631 | Wavelet-HHL - Histogram - Variance | 666 | Hog-1_0_1_8 |

**Supplementary Table 1 (continued)**

| No. | Feature name | No. | Feature name |
| --- | --- | --- | --- |
| 667 | Hog-2_0_1_8 | 707 | Hog-0_0_2_15 |
| 668 | Hog-1_2_1_8 | 708 | Hog-0_1_2_15 |
| 669 | Hog-1_1_2_8 | 709 | Hog-0_2_2_15 |
| 670 | Hog-1_0_0_9 | 710 | Hog-0_0_0_16 |
| 671 | Hog-2_2_0_9 | 711 | Hog-0_2_0_16 |
| 672 | Hog-2_0_1_9 | 712 | Hog-0_0_1_16 |
| 673 | Hog-2_1_1_9 | 713 | Hog-2_0_1_16 |
| 674 | Hog-1_2_1_9 | 714 | Hog-0_0_0_18 |
| 675 | Hog-0_0_2_9 | 715 | Hog-1_0_0_18 |
| 676 | Hog-1_1_2_9 | 716 | Hog-2_1_0_18 |
| 677 | Hog-2_1_2_9 | 717 | Hog-0_2_0_18 |
| 678 | Hog-0_0_0_10 | 718 | Hog-1_2_0_18 |
| 679 | Hog-0_0_1_10 | 719 | Hog-2_2_0_18 |
| 680 | Hog-2_0_1_10 | 720 | Hog-1_0_1_18 |
| 681 | Hog-0_0_2_10 | 721 | Hog-2_0_1_18 |
| 682 | Hog-0_2_2_10 | 722 | Hog-1_1_1_18 |
| 683 | Hog-2_0_0_11 | 723 | Hog-2_1_1_18 |
| 684 | Hog-0_1_0_11 | 724 | Hog-0_2_1_18 |
| 685 | Hog-2_0_2_11 | 725 | Hog-0_0_2_18 |
| 686 | Hog-2_1_0_12 | 726 | Hog-2_0_2_18 |
| 687 | Hog-2_2_0_12 | 727 | Hog-1_1_2_18 |
| 688 | Hog-2_1_1_12 | 728 | Hog-2_1_2_18 |
| 689 | Hog-1_1_2_12 | 729 | Hog-0_1_0_19 |
| 690 | Hog-0_2_2_12 | 730 | Hog-2_1_0_19 |
| 691 | Hog-0_0_0_13 | 731 | Hog-1_2_0_19 |
| 692 | Hog-0_1_0_13 | 732 | Hog-1_0_1_19 |
| 693 | Hog-1_1_0_13 | 733 | Hog-2_0_1_19 |
| 694 | Hog-0_2_0_13 | 734 | Hog-1_1_1_19 |
| 695 | Hog-2_1_1_13 | 735 | Hog-1_1_2_19 |
| 696 | Hog-0_0_2_13 | 736 | Statistics-[0, 1, 0]-Contrast |
| 697 | Hog-0_1_2_13 | 737 | Statistics-[0, 1, 0]-Variance |
| 698 | Hog-1_1_2_13 | 738 | Statistics-[0, 1, 0]-Inertia |
| 699 | Hog-2_1_2_13 | 739 | Statistics-[0, 1, 0]-Inverse Variance |
| 700 | Hog-2_2_2_13 | 740 | Statistics-[-1, 1, 0]-Contrast |
| 701 | Hog-2_1_0_14 | 741 | Statistics-[-1, 1, 0]-Variance |
| 702 | Hog-2_2_0_14 | 742 | Statistics-[-1, 1, 0]-Inertia |
| 703 | Hog-0_0_2_14 | 743 | Statistics-[-1, 0, 0]-Variance |
| 704 | Hog-0_1_2_14 | 744 | Statistics-[-1, 0, 0]-Cluster Tendency |
| 705 | Hog-0_2_2_14 | 745 | Statistics-[-1, -1, 0]-Contrast |
| 706 | Hog-2_0_0_15 | 746 | Statistics-[-1, -1, 0]-Variance |

**Supplementary Table 1 (continued)**

| No. | Feature name | No. | Feature name |
| --- | --- | --- | --- |
| 747 | Statistics-[-1, -1, 0]-Inertia | 763 | Statistics-[-1, 0, -1]-Cluster Tendency |
| 748 | Statistics-[0, 1, -1]-Contrast | 764 | Statistics-[1, 0, -1]-Variance |
| 749 | Statistics-[0, 1, -1]-Variance | 765 | Statistics-[1, 0, -1]-Cluster Tendency |
| 750 | Statistics-[0, 1, -1]-Inertia | 766 | Statistics-[-1, 1, -1]-Contrast |
| 751 | Statistics-[0, 1, -1]-Inverse Variance | 767 | Statistics-[-1, 1, -1]-Variance |
| 752 | Statistics-[0, 0, -1]-Entropy | 768 | Statistics-[-1, 1, -1]-Inertia |
| 753 | Statistics-[0, 0, -1]-Contrast | 769 | Statistics-[1, -1, -1]-Contrast |
| 754 | Statistics-[0, 0, -1]-Variance | 770 | Statistics-[1, -1, -1]-Variance |
| 755 | Statistics-[0, 0, -1]-Inertia | 771 | Statistics-[1, -1, -1]-Inertia |
| 756 | Statistics-[0, 0, -1]-Cluster Tendency | 772 | Statistics-[-1, -1, -1]-Contrast |
| 757 | Statistics-[0, 0, -1]-Inverse Variance | 773 | Statistics-[-1, -1, -1]-Variance |
| 758 | Statistics-[0, -1, -1]-Contrast | 774 | Statistics-[-1, -1, -1]-Inertia |
| 759 | Statistics-[0, -1, -1]-Variance | 775 | Statistics-[1, 1, -1]-Contrast |
| 760 | Statistics-[0, -1, -1]-Inertia | 776 | Statistics-[1, 1, -1]-Variance |
| 761 | Statistics-[0, -1, -1]-Inverse Variance | 777 | Statistics-[1, 1, -1]-Inertia |
| 762 | Statistics-[-1, 0, -1]-Variance |  |  |

**Supplementary Table 1 (continued)**

Abbreviations: MRI, magnetic resonance imaging; GLZSM, grey level zone size matrix; Hog, histogram of oriented gradient; GTSDM, grey tone spatial dependence matrix.
