# Supplementary material for: Risk stratification and prognostic value of multi-modal MRI-based radiomics for extranodal nasal-type NK/T-cell lymphoma
Source: BMC Cancer. 2023 Jan 25;23:88. doi: 10.1186/s12885-023-10557-3 (PMC9878926; doi:10.1186/s12885-023-10557-3)
Supplement: Supplementary file 2 — Supplementary Material 2 [file 12885_2023_10557_MOESM2_ESM.docx]

**Supplementary Table 2.** The mean and standard deviation of the multi-modal MRI radiomics features.

| Feature name | Mean | Standard deviation |
| --- | --- | --- |
| Texture-GLZSM-Large Zone / High Gray Emphasis | 0.006135870 | 0.078331791 |
| Texture-GLZSM-Gray-Level Non-Uniformity | 0.014196161 | 0.119147642 |
| Texture-GLZSM-Zone Size Non-Uniformity | 0.023771348 | 0.154179596 |
| Wavelet-LLL - Histogram - Mean | 0.036031179 | 0.189818805 |
| Wavelet-LLL - Histogram - Energy | 0.044209075 | 0.210259542 |
| Wavelet-LLL - Histogram - Entropy | 0.038854322 | 0.197114996 |
| Wavelet-LLH - Histogram - Variance | 0.023998945 | 0.154915930 |
| Wavelet-LLH - Histogram - Kurtosis | 0.029576976 | 0.171979580 |
| Wavelet-LLH - Histogram - Energy | 0.060113404 | 0.245180351 |
| Wavelet-LLH - Histogram - Entropy | 0.039895797 | 0.199739322 |
| Wavelet-LHL - Histogram - Variance | 0.012793102 | 0.113106595 |
| Wavelet-LHL - Histogram - Skewness | 0.035109030 | 0.187374038 |
| Wavelet-LHL - Histogram - Entropy | 0.036382592 | 0.190742213 |
| Wavelet-LHH - Histogram - Skewness | 0.017174507 | 0.131051543 |
| Hog-2_0_0_0 | 0.019678654 | 0.140280627 |
| Hog-0_1_0_0 | 0.007941584 | 0.089115567 |
| Hog-1_1_0_0 | 0.040212651 | 0.200530923 |
| Hog-2_1_0_0 | 0.033771236 | 0.183769520 |
| Hog-0_2_0_0 | 0.007517321 | 0.086702487 |
| Hog-0_0_1_0 | 0.015466342 | 0.124363748 |
| Hog-2_0_1_0 | 0.019531048 | 0.139753527 |
| Hog-0_1_1_0 | 0.017203914 | 0.131163693 |
| Hog-2_1_1_0 | 0.012951930 | 0.113806546 |
| Hog-1_2_1_0 | 0.012943437 | 0.113769226 |
| Hog-2_0_2_0 | 0.022665367 | 0.150550215 |
| Hog-0_0_0_1 | 0.017909221 | 0.133825337 |
| Hog-1_0_0_1 | 0.019567515 | 0.139883934 |
| Hog-2_0_0_1 | 0.012467221 | 0.111656711 |
| Hog-0_1_0_1 | 0.028353614 | 0.168385315 |
| Hog-1_1_0_1 | 0.014728520 | 0.121361114 |
| Hog-0_1_1_1 | 0.020863203 | 0.144441000 |
| Hog-1_1_2_1 | 0.022009336 | 0.148355438 |
| Hog-0_0_0_2 | 0.034894625 | 0.186801031 |
| Hog-1_1_0_2 | 0.010479949 | 0.102371622 |
| Hog-2_2_0_2 | 0.032026113 | 0.178958412 |
| Hog-0_0_1_2 | 0.024987139 | 0.158073209 |
| Hog-1_0_1_2 | 0.018893543 | 0.137453786 |
| Hog-0_2_1_2 | 0.014177441 | 0.119069061 |
| Hog-1_0_0_3 | 0.025105095 | 0.158445873 |
| Hog-0_1_0_3 | 0.035840679 | 0.189316346 |
| Hog-1_1_0_3 | 0.013896418 | 0.117883069 |
| Hog-2_1_0_3 | 0.008839821 | 0.094020322 |
| Hog-2_2_0_3 | 0.024718266 | 0.157220437 |
| Hog-0_1_1_3 | 0.030016870 | 0.173253774 |
| Hog-1_0_2_3 | 0.011927138 | 0.109211439 |
| Hog-0_0_0_4 | 0.012070504 | 0.109865845 |
| Hog-1_2_0_4 | 0.017376631 | 0.131820449 |
| Hog-0_0_1_4 | 0.010648462 | 0.103191385 |
| Hog-2_0_0_5 | 0.024054736 | 0.155095892 |
| Hog-0_1_0_5 | 0.011463080 | 0.107065774 |
| Hog-1_0_2_5 | 0.020901966 | 0.144575122 |
| Hog-0_0_0_6 | 0.008449399 | 0.091920610 |
| Hog-0_0_1_6 | 0.022900224 | 0.151328199 |
| Hog-0_0_2_6 | 0.007960816 | 0.089223404 |
| Hog-2_1_0_7 | 0.016872216 | 0.129893095 |
| Hog-0_2_0_7 | 0.013183171 | 0.114817992 |
| Hog-2_2_0_7 | 0.022229378 | 0.149095198 |
| Hog-0_1_1_7 | 0.030552049 | 0.174791443 |
| Hog-2_1_1_7 | 0.022334635 | 0.149447767 |
| Hog-0_2_1_7 | 0.010423717 | 0.102096607 |
| Hog-2_1_2_7 | 0.013686076 | 0.116987504 |
| Hog-0_2_2_7 | 0.025245977 | 0.158889825 |
| Hog-2_0_0_8 | 0.014839435 | 0.121817219 |
| Hog-0_1_0_8 | 0.025223089 | 0.158817787 |
| Hog-1_1_0_8 | 0.018236208 | 0.135041504 |
| Hog-2_1_0_8 | 0.008221956 | 0.090675000 |
| Hog-0_2_0_8 | 0.016608490 | 0.128873932 |
| Hog-1_2_0_8 | 0.021140357 | 0.145397237 |
| Hog-2_2_0_8 | 0.006642751 | 0.081503076 |
| Hog-2_0_1_8 | 0.018924995 | 0.137568148 |
| Hog-2_1_1_8 | 0.024752759 | 0.157330097 |
| Hog-1_2_1_8 | 0.014812837 | 0.121708001 |
| Hog-1_0_2_8 | 0.017237211 | 0.131290561 |
| Hog-0_0_0_9 | 0.006552323 | 0.080946421 |
| Hog-2_0_0_9 | 0.012042780 | 0.109739600 |
| Hog-0_1_0_9 | 0.026784590 | 0.163659981 |
| Hog-1_1_0_9 | 0.013155645 | 0.114698062 |
| Hog-2_1_0_9 | 0.015038737 | 0.122632527 |
| Hog-2_2_0_9 | 0.006715523 | 0.081948292 |
| Hog-2_0_1_9 | 0.019189766 | 0.138527132 |
| Hog-1_1_1_9 | 0.022088135 | 0.148620776 |
| Hog-2_1_1_9 | 0.009511054 | 0.097524633 |
| Hog-0_2_1_9 | 0.009543349 | 0.097690067 |
| Hog-0_0_2_9 | 0.014972369 | 0.122361634 |
| Hog-1_0_0_10 | 0.015757319 | 0.125528160 |
| Hog-2_0_0_10 | 0.026618638 | 0.163152193 |
| Hog-2_1_0_10 | 0.012815797 | 0.113206876 |
| Hog-1_0_1_10 | 0.017188277 | 0.131104071 |
| Hog-2_0_1_10 | 0.011191437 | 0.105789590 |
| Hog-2_1_1_10 | 0.018766129 | 0.136989521 |
| Hog-1_0_2_10 | 0.012322630 | 0.111007341 |
| Hog-2_0_2_10 | 0.027134709 | 0.164726165 |
| Hog-0_2_2_10 | 0.013036516 | 0.114177565 |
| Hog-0_0_0_11 | 0.007887667 | 0.088812538 |
| Hog-1_0_0_11 | 0.022626104 | 0.150419760 |
| Hog-2_0_0_11 | 0.011245694 | 0.106045718 |
| Hog-1_1_0_11 | 0.010631837 | 0.103110797 |
| Hog-2_1_0_11 | 0.027729117 | 0.166520621 |
| Hog-0_0_1_11 | 0.015108902 | 0.122918275 |
| Hog-1_0_1_11 | 0.017811683 | 0.133460418 |
| Hog-2_0_1_11 | 0.007519495 | 0.086715019 |
| Hog-0_1_1_11 | 0.007140645 | 0.084502339 |
| Hog-1_1_1_11 | 0.034710099 | 0.186306466 |
| Hog-0_0_2_11 | 0.015313734 | 0.123748674 |
| Hog-1_0_2_11 | 0.014563768 | 0.120680438 |
| Hog-2_1_0_12 | 0.013518784 | 0.116270305 |
| Hog-2_2_0_12 | 0.027737995 | 0.166547276 |
| Hog-0_2_1_12 | 0.008723528 | 0.093399827 |
| Hog-2_1_2_12 | 0.016374598 | 0.127963268 |
| Hog-0_2_2_12 | 0.009345072 | 0.096669915 |
| Hog-0_0_0_13 | 0.015843120 | 0.125869455 |
| Hog-1_1_0_13 | 0.010396522 | 0.101963336 |
| Hog-2_1_0_13 | 0.018086358 | 0.134485530 |
| Hog-1_2_0_13 | 0.031603505 | 0.177773747 |
| Hog-2_2_0_13 | 0.015524448 | 0.124597141 |
| Hog-0_2_1_13 | 0.012701644 | 0.112701569 |
| Hog-2_0_2_13 | 0.013241204 | 0.115070431 |
| Hog-2_0_0_14 | 0.034372110 | 0.185397169 |
| Hog-0_0_2_14 | 0.021006301 | 0.144935505 |
| Hog-0_1_2_14 | 0.017363773 | 0.131771672 |
| Hog-0_2_2_14 | 0.017997413 | 0.134154439 |
| Hog-2_0_0_15 | 0.019843161 | 0.140865757 |
| Hog-0_0_2_15 | 0.014906469 | 0.122092052 |
| Hog-0_1_2_15 | 0.011674242 | 0.108047404 |
| Hog-1_0_0_16 | 0.025004215 | 0.158127212 |
| Hog-2_1_1_16 | 0.013529831 | 0.116317800 |
| Hog-2_0_2_16 | 0.011816910 | 0.108705610 |
| Hog-0_0_0_17 | 0.005920002 | 0.076941551 |
| Hog-2_1_0_17 | 0.019477843 | 0.139563042 |
| Hog-2_2_0_17 | 0.035698181 | 0.188939623 |
| Hog-0_0_1_17 | 0.015861519 | 0.125942524 |
| Hog-2_1_1_17 | 0.022672114 | 0.150572620 |
| Hog-0_0_2_17 | 0.007223129 | 0.084988991 |
| Hog-2_1_2_17 | 0.016458737 | 0.128291610 |
| Hog-2_0_0_18 | 0.008427022 | 0.091798811 |
| Hog-0_1_0_18 | 0.008363310 | 0.091451135 |
| Hog-1_1_0_18 | 0.018039806 | 0.134312345 |
| Hog-2_1_0_18 | 0.010945023 | 0.104618462 |
| Hog-2_2_0_18 | 0.006404235 | 0.080026467 |
| Hog-2_0_1_18 | 0.028866275 | 0.169900779 |
| Hog-1_1_1_18 | 0.027838676 | 0.166849262 |
| Hog-2_2_1_18 | 0.010241857 | 0.101202058 |
| Hog-1_0_2_18 | 0.023591964 | 0.153596758 |
| Hog-0_0_0_19 | 0.019469368 | 0.139532679 |
| Hog-0_1_0_19 | 0.017900274 | 0.133791907 |
| Hog-1_1_0_19 | 0.018606982 | 0.136407412 |
| Hog-2_1_0_19 | 0.027783192 | 0.166682908 |
| Hog-0_2_0_19 | 0.018870452 | 0.137369765 |
| Hog-0_0_1_19 | 0.009146832 | 0.095639069 |
| Hog-1_0_1_19 | 0.016257254 | 0.127503938 |
| Hog-2_0_1_19 | 0.019538094 | 0.139778731 |
| Hog-2_1_1_19 | 0.006897483 | 0.083051088 |
| Hog-0_2_1_19 | 0.016405067 | 0.128082265 |
| Statistics-[0, 1, 0]-Contrast | 0.015427569 | 0.124207765 |
| Statistics-[0, 1, 0]-Inertia | 0.015427569 | 0.124207765 |
| Statistics-[0, 1, 0]-Inverse Variance | 0.050495063 | 0.224711067 |
| Statistics-[-1, 1, 0]-Contrast | 0.023058093 | 0.151848914 |
| Statistics-[-1, 1, 0]-Inertia | 0.023058093 | 0.151848914 |
| Statistics-[-1, -1, 0]-Contrast | 0.017953957 | 0.133992377 |
| Statistics-[-1, -1, 0]-Inertia | 0.017953957 | 0.133992377 |
| Statistics-[0, 1, -1]-Contrast | 0.023101154 | 0.151990637 |
| Statistics-[0, 1, -1]-Inertia | 0.023101154 | 0.151990637 |
| Statistics-[0, 1, -1]-Inverse Variance | 0.047589319 | 0.218149763 |
| Statistics-[0, 0, -1]-Contrast | 0.027132003 | 0.164717949 |
| Statistics-[0, 0, -1]-Inertia | 0.027132003 | 0.164717949 |
| Statistics-[0, 0, -1]-Inverse Variance | 0.041632571 | 0.204040611 |
| Statistics-[0, -1, -1]-Contrast | 0.014169670 | 0.119036424 |
| Statistics-[0, -1, -1]-Inertia | 0.014169670 | 0.119036424 |
| Statistics-[0, -1, -1]-Inverse Variance | 0.047793287 | 0.218616757 |
| Statistics-[-1, 1, -1]-Contrast | 0.031678856 | 0.177985549 |
| Statistics-[-1, 1, -1]-Inertia | 0.031678856 | 0.177985549 |
| Statistics-[1, -1, -1]-Contrast | 0.019636767 | 0.140131250 |
| Statistics-[1, -1, -1]-Inertia | 0.019636767 | 0.140131250 |
| Statistics-[-1, -1, -1]-Contrast | 0.015941875 | 0.126261140 |
| Statistics-[-1, -1, -1]-Inertia | 0.015941875 | 0.126261140 |
| Statistics-[1, 1, -1]-Contrast | 0.025709635 | 0.160342242 |
| Statistics-[1, 1, -1]-Inertia | 0.025709635 | 0.160342242 |
| (2) Texture-Histogram-Variance | 0.035027387 | 0.187156051 |
| (3) Texture-Histogram-Skewness | 0.019501571 | 0.139648024 |
| (4) Texture-Histogram-Kurtosis | 0.008575688 | 0.092605009 |
| (5) Texture-Histogram-Energy | 0.021663537 | 0.147185382 |
| (6) Texture-Histogram-Entropy | 0.024498418 | 0.156519704 |
| (14) Texture-GTSDM-Sum Entropy | 0.024089292 | 0.155207254 |
| (15) Texture-GTSDM-Entropy | 0.027023437 | 0.164388069 |
| (17) Texture-GTSDM-Difference Entropy | 0.035378010 | 0.188090430 |
| (19) Texture-GTSDM-Information Correlation 2 | 0.044383892 | 0.210674850 |
| (32) Texture-GLZSM-Small Zone Size Emphasis | 0.029270419 | 0.171085998 |
| (36) Texture-GLZSM-Small Zone / Low Gray Emphasis | 0.026539917 | 0.162910764 |
| (39) Texture-GLZSM-Large Zone / High Gray Emphasis | 0.024506199 | 0.156544558 |
| (40) Texture-GLZSM-Gray-Level Non-Uniformity | 0.011364875 | 0.106606166 |
| (41) Texture-GLZSM-Zone Size Non-Uniformity | 0.016146222 | 0.127067786 |
| (42) Texture-GLZSM-Zone Size Percentage | 0.034798088 | 0.186542458 |
| Wavelet-LLL - Histogram - Variance | 0.032831735 | 0.181195296 |
| Wavelet-LLL - Histogram - Kurtosis | 0.018085652 | 0.134482907 |
| Wavelet-LLL - Histogram - Energy | 0.028866076 | 0.169900194 |
| Wavelet-LLL - Histogram - Entropy | 0.031716810 | 0.178092138 |
| Wavelet-LLH - Histogram - Variance | 0.030954695 | 0.175939464 |
| Wavelet-LHL - Histogram - Variance | 0.028428679 | 0.168608065 |
| Wavelet-LHL - Histogram - Skewness | 0.064250366 | 0.253476558 |
| Wavelet-LHL - Histogram - Kurtosis | 0.039502351 | 0.198751985 |
| Wavelet-LHL - Histogram - Energy | 0.040119910 | 0.200299552 |
| Wavelet-LHL - Histogram - Entropy | 0.041863830 | 0.204606525 |
| Wavelet-LHH - Histogram - Variance | 0.018876051 | 0.137390141 |
| Wavelet-HLL - Histogram - Variance | 0.025497732 | 0.159680092 |
| Wavelet-HLL - Histogram - Energy | 0.045785765 | 0.213976084 |
| Wavelet-HLL - Histogram - Entropy | 0.041326587 | 0.203289416 |
| Wavelet-HLH - Histogram - Variance | 0.022237476 | 0.149122352 |
| Wavelet-HHL - Histogram - Variance | 0.025780717 | 0.160563746 |
| Wavelet-HHL - Histogram - Kurtosis | 0.017636742 | 0.132803395 |
| Wavelet-HHL - Histogram - Energy | 0.032286889 | 0.179685528 |
| Wavelet-HHL - Histogram - Entropy | 0.037078060 | 0.192556641 |
| Wavelet-HHH - Histogram - Variance | 0.015111132 | 0.122927345 |
| Hog-1_0_0_0 | 0.008272882 | 0.090955384 |
| Hog-2_0_0_0 | 0.011159617 | 0.105639087 |
| Hog-2_1_0_0 | 0.020090964 | 0.141742599 |
| Hog-0_2_0_0 | 0.025526873 | 0.159771315 |
| Hog-0_2_1_0 | 0.035230295 | 0.187697350 |
| Hog-2_0_2_0 | 0.025965073 | 0.161136813 |
| Hog-0_0_0_1 | 0.013294453 | 0.115301575 |
| Hog-2_0_0_1 | 0.020371574 | 0.142729022 |
| Hog-0_1_0_1 | 0.019989454 | 0.141384065 |
| Hog-2_1_2_1 | 0.022802437 | 0.151004758 |
| Hog-0_0_0_2 | 0.016969181 | 0.130265811 |
| Hog-2_0_0_2 | 0.021849918 | 0.147817176 |
| Hog-1_1_0_2 | 0.026931531 | 0.164108292 |
| Hog-0_2_0_2 | 0.011528363 | 0.107370215 |
| Hog-2_2_0_2 | 0.017702932 | 0.133052368 |
| Hog-2_1_0_3 | 0.010290113 | 0.101440194 |
| Hog-1_2_0_3 | 0.009245615 | 0.096154123 |
| Hog-2_2_0_3 | 0.020120329 | 0.141846147 |
| Hog-2_1_2_3 | 0.009113798 | 0.095466214 |
| Hog-2_2_2_3 | 0.024246071 | 0.155711499 |
| Hog-0_0_0_4 | 0.009695057 | 0.098463479 |
| Hog-2_0_0_4 | 0.019184229 | 0.138507145 |
| Hog-0_2_0_4 | 0.010310363 | 0.101539959 |
| Hog-2_2_2_4 | 0.039306751 | 0.198259302 |
| Hog-0_1_0_5 | 0.012695362 | 0.112673696 |
| Hog-2_0_1_5 | 0.019574907 | 0.139910355 |
| Hog-0_0_2_5 | 0.025424099 | 0.159449362 |
| Hog-0_0_0_6 | 0.019552106 | 0.139828844 |
| Hog-2_1_0_6 | 0.015733930 | 0.125434962 |
| Hog-0_0_0_7 | 0.011223221 | 0.105939703 |
| Hog-2_2_0_7 | 0.016807049 | 0.129642004 |
| Hog-0_0_1_7 | 0.024092538 | 0.155217712 |
| Hog-0_2_2_7 | 0.018640324 | 0.136529572 |
| Hog-1_0_0_8 | 0.021256057 | 0.145794571 |
| Hog-2_0_0_8 | 0.032928101 | 0.181461019 |
| Hog-0_1_0_8 | 0.023504101 | 0.153310473 |
| Hog-2_1_0_8 | 0.016149127 | 0.127079215 |
| Hog-0_2_0_8 | 0.009798546 | 0.098987605 |
| Hog-1_0_1_8 | 0.013669120 | 0.116915014 |
| Hog-1_1_1_8 | 0.019831209 | 0.140823325 |
| Hog-2_0_2_8 | 0.018974707 | 0.137748709 |
| Hog-1_1_2_8 | 0.012295775 | 0.110886316 |
| Hog-2_1_2_8 | 0.043759648 | 0.209188068 |
| Hog-1_0_0_9 | 0.025396489 | 0.159362760 |
| Hog-1_1_0_9 | 0.021455762 | 0.146477857 |
| Hog-0_2_0_9 | 0.009488982 | 0.097411404 |
| Hog-1_0_1_9 | 0.013435296 | 0.115910724 |
| Hog-1_1_1_9 | 0.019379091 | 0.139208805 |
| Hog-2_1_1_9 | 0.015846386 | 0.125882429 |
| Hog-1_2_1_9 | 0.035583601 | 0.188636161 |
| Hog-2_0_2_9 | 0.028479740 | 0.168759415 |
| Hog-1_1_2_9 | 0.025155170 | 0.158603815 |
| Hog-1_2_2_9 | 0.035900825 | 0.189475130 |
| Hog-0_0_0_10 | 0.015249811 | 0.123490125 |
| Hog-1_0_0_10 | 0.009435093 | 0.097134408 |
| Hog-2_0_0_10 | 0.026663597 | 0.163289918 |
| Hog-0_1_1_10 | 0.010158112 | 0.100787459 |
| Hog-0_0_2_10 | 0.011452670 | 0.107017147 |
| Hog-1_0_2_10 | 0.024633402 | 0.156950317 |
| Hog-2_0_2_10 | 0.025357921 | 0.159241706 |
| Hog-1_0_0_11 | 0.027446280 | 0.165669188 |
| Hog-1_0_1_11 | 0.023302368 | 0.152651132 |
| Hog-2_0_1_11 | 0.035961398 | 0.189634907 |
| Hog-2_1_1_11 | 0.020244081 | 0.142281697 |
| Hog-2_0_2_11 | 0.027132904 | 0.164720684 |
| Hog-0_2_2_11 | 0.017600482 | 0.132666808 |
| Hog-1_2_0_12 | 0.024478134 | 0.156454896 |
| Hog-2_2_0_12 | 0.019286018 | 0.138874107 |
| Hog-0_0_2_12 | 0.011361515 | 0.106590410 |
| Hog-2_1_2_12 | 0.046589758 | 0.215846608 |
| Hog-0_2_2_12 | 0.025130011 | 0.158524480 |
| Hog-2_0_0_13 | 0.025614956 | 0.160046731 |
| Hog-0_1_0_13 | 0.020998743 | 0.144909432 |
| Hog-1_1_0_13 | 0.021371919 | 0.146191378 |
| Hog-2_2_0_13 | 0.015575305 | 0.124801060 |
| Hog-1_0_1_13 | 0.040085726 | 0.200214199 |
| Hog-2_0_2_13 | 0.024594933 | 0.156827719 |
| Hog-0_1_2_13 | 0.024757962 | 0.157346631 |
| Hog-2_2_2_13 | 0.025875442 | 0.160858454 |
| Hog-2_0_1_14 | 0.012375987 | 0.111247413 |
| Hog-0_0_2_14 | 0.009683501 | 0.098404779 |
| Hog-2_1_2_14 | 0.043134455 | 0.207688360 |
| Hog-0_2_2_14 | 0.021603660 | 0.146981835 |
| Hog-2_1_0_15 | 0.028756028 | 0.169576024 |
| Hog-0_0_2_15 | 0.011618769 | 0.107790392 |
| Hog-0_1_2_15 | 0.024269652 | 0.155787200 |
| Hog-0_0_0_16 | 0.007321239 | 0.085564238 |
| Hog-2_0_0_16 | 0.010024636 | 0.100123106 |
| Hog-2_0_1_16 | 0.024834613 | 0.157590015 |
| Hog-0_0_2_16 | 0.023969882 | 0.154822098 |
| Hog-2_2_0_17 | 0.011743721 | 0.108368452 |
| Hog-2_0_1_17 | 0.035089477 | 0.187321853 |
| Hog-1_1_0_18 | 0.012754601 | 0.112936272 |
| Hog-1_0_1_18 | 0.018750540 | 0.136932610 |
| Hog-0_1_1_18 | 0.009556539 | 0.097757552 |
| Hog-0_0_2_18 | 0.011284795 | 0.106229918 |
| Hog-1_0_2_18 | 0.014794521 | 0.121632728 |
| Hog-1_1_0_19 | 0.009852382 | 0.099259167 |
| Hog-0_2_0_19 | 0.024450479 | 0.156366489 |
| Hog-2_2_0_19 | 0.029783398 | 0.172578671 |
| Hog-1_0_1_19 | 0.010918860 | 0.104493351 |
| Hog-1_1_1_19 | 0.018965585 | 0.137715593 |
| Hog-2_1_1_19 | 0.017597155 | 0.132654270 |
| Hog-0_0_2_19 | 0.008575782 | 0.092605517 |
| Hog-1_0_2_19 | 0.011814468 | 0.108694377 |
| Hog-2_0_2_19 | 0.034394491 | 0.185457519 |
| Hog-1_1_2_19 | 0.015471775 | 0.124385588 |
| Hog-1_2_2_19 | 0.023652696 | 0.153794332 |
| Statistics-[0, 1, 0]-Entropy | 0.040045033 | 0.200112550 |
| Statistics-[0, 1, 0]-Contrast | 0.041911762 | 0.204723623 |
| Statistics-[0, 1, 0]-Variance | 0.041027963 | 0.202553604 |
| Statistics-[0, 1, 0]-Inertia | 0.041911762 | 0.204723623 |
| Statistics-[0, 1, 0]-Cluster Shade | 0.020660418 | 0.143737322 |
| Statistics-[0, 1, 0]-Cluster Tendency | 0.046565791 | 0.215791081 |
| Statistics-[0, 1, 0]-Inverse Variance | 0.057460224 | 0.239708622 |
| Statistics-[-1, 1, 0]-Contrast | 0.041101140 | 0.202734162 |
| Statistics-[-1, 1, 0]-Variance | 0.040861975 | 0.202143452 |
| Statistics-[-1, 1, 0]-Inertia | 0.041101140 | 0.202734162 |
| Statistics-[-1, 1, 0]-Cluster Shade | 0.020362864 | 0.142698506 |
| Statistics-[-1, 1, 0]-Cluster Tendency | 0.047199778 | 0.217255098 |
| Statistics-[-1, 0, 0]-Variance | 0.040893900 | 0.202222402 |
| Statistics-[-1, 0, 0]-Cluster Shade | 0.021463797 | 0.146505278 |
| Statistics-[-1, 0, 0]-Cluster Tendency | 0.047532117 | 0.218018615 |
| Statistics-[-1, -1, 0]-Contrast | 0.037835708 | 0.194514031 |
| Statistics-[-1, -1, 0]-Variance | 0.040863102 | 0.202146239 |
| Statistics-[-1, -1, 0]-Inertia | 0.037835708 | 0.194514031 |
| Statistics-[-1, -1, 0]-Cluster Shade | 0.019691162 | 0.140325200 |
| Statistics-[-1, -1, 0]-Cluster Tendency | 0.044982082 | 0.212089798 |
| Statistics-[0, 1, -1]-Entropy | 0.039102610 | 0.197743800 |
| Statistics-[0, 1, -1]-Contrast | 0.042980074 | 0.207316361 |
| Statistics-[0, 1, -1]-Variance | 0.041328358 | 0.203293773 |
| Statistics-[0, 1, -1]-Inertia | 0.042980074 | 0.207316361 |
| Statistics-[0, 1, -1]-Cluster Shade | 0.021065666 | 0.145140159 |
| Statistics-[0, 1, -1]-Cluster Tendency | 0.045606493 | 0.213556768 |
| Statistics-[0, 1, -1]-Inverse Variance | 0.056160734 | 0.236982560 |
| Statistics-[0, 0, -1]-Entropy | 0.037992411 | 0.194916421 |
| Statistics-[0, 0, -1]-Contrast | 0.046284904 | 0.215139267 |
| Statistics-[0, 0, -1]-Variance | 0.041325038 | 0.203285607 |
| Statistics-[0, 0, -1]-Inertia | 0.046284904 | 0.215139267 |
| Statistics-[0, 0, -1]-Cluster Shade | 0.022068128 | 0.148553451 |
| Statistics-[0, 0, -1]-Cluster Tendency | 0.047845512 | 0.218736171 |
| Statistics-[0, 0, -1]-Inverse Variance | 0.058426714 | 0.241716185 |
| Statistics-[0, -1, -1]-Entropy | 0.040366049 | 0.200913039 |
| Statistics-[0, -1, -1]-Contrast | 0.042660908 | 0.206545171 |
| Statistics-[0, -1, -1]-Variance | 0.041268092 | 0.203145495 |
| Statistics-[0, -1, -1]-Inertia | 0.042660908 | 0.206545171 |
| Statistics-[0, -1, -1]-Cluster Shade | 0.020466331 | 0.143060585 |
| Statistics-[0, -1, -1]-Cluster Tendency | 0.044478495 | 0.210899253 |
| Statistics-[0, -1, -1]-Inverse Variance | 0.056137688 | 0.236933931 |
| Statistics-[-1, 0, -1]-Variance | 0.041176611 | 0.202920209 |
| Statistics-[-1, 0, -1]-Cluster Shade | 0.021587661 | 0.146927401 |
| Statistics-[-1, 0, -1]-Cluster Tendency | 0.047524841 | 0.218001929 |
| Statistics-[1, 0, -1]-Variance | 0.041152030 | 0.202859631 |
| Statistics-[1, 0, -1]-Cluster Shade | 0.021256977 | 0.145797726 |
| Statistics-[1, 0, -1]-Cluster Tendency | 0.048429190 | 0.220066332 |
| Statistics-[-1, 1, -1]-Contrast | 0.040363957 | 0.200907833 |
| Statistics-[-1, 1, -1]-Variance | 0.041171241 | 0.202906976 |
| Statistics-[-1, 1, -1]-Inertia | 0.040363957 | 0.200907833 |
| Statistics-[-1, 1, -1]-Cluster Shade | 0.020931273 | 0.144676444 |
| Statistics-[-1, 1, -1]-Cluster Tendency | 0.047733951 | 0.218481009 |
| Statistics-[1, -1, -1]-Contrast | 0.043583365 | 0.208766293 |
| Statistics-[1, -1, -1]-Variance | 0.041085735 | 0.202696165 |
| Statistics-[1, -1, -1]-Inertia | 0.043583365 | 0.208766293 |
| Statistics-[1, -1, -1]-Cluster Shade | 0.020090522 | 0.141741038 |
| Statistics-[1, -1, -1]-Cluster Tendency | 0.046187681 | 0.214913195 |
| Statistics-[-1, -1, -1]-Contrast | 0.038556746 | 0.196358718 |
| Statistics-[-1, -1, -1]-Variance | 0.041112460 | 0.202762077 |
| Statistics-[-1, -1, -1]-Inertia | 0.038556746 | 0.196358718 |
| Statistics-[-1, -1, -1]-Cluster Shade | 0.019639785 | 0.140142018 |
| Statistics-[-1, -1, -1]-Cluster Tendency | 0.045069587 | 0.212295988 |
| Statistics-[-1, -1, -1]-Inverse Variance | 0.047191669 | 0.217236435 |
| Statistics-[1, 1, -1]-Contrast | 0.038487985 | 0.196183549 |
| Statistics-[1, 1, -1]-Variance | 0.041147431 | 0.202848296 |
| Statistics-[1, 1, -1]-Inertia | 0.038487985 | 0.196183549 |
| Statistics-[1, 1, -1]-Cluster Shade | 0.019978722 | 0.141346108 |
| Statistics-[1, 1, -1]-Cluster Tendency | 0.045310639 | 0.212862958 |
| Statistics-[1, 1, -1]-Inverse Variance | 0.045582038 | 0.213499504 |
| (3) Texture-Histogram-Skewness | 0.040628933 | 0.201566200 |
| (4) Texture-Histogram-Kurtosis | 0.034493815 | 0.185725106 |
| (5) Texture-Histogram-Energy | 0.023499415 | 0.153295190 |
| (6) Texture-Histogram-Entropy | 0.031620091 | 0.177820390 |
| (14) Texture-GTSDM-Sum Entropy | 0.034497212 | 0.185734250 |
| (15) Texture-GTSDM-Entropy | 0.031557883 | 0.177645385 |
| (17) Texture-GTSDM-Difference Entropy | 0.027289132 | 0.165194224 |
| (32) Texture-GLZSM-Small Zone Size Emphasis | 0.035280262 | 0.187830409 |
| (36) Texture-GLZSM-Small Zone / Low Gray Emphasis | 0.052249528 | 0.228581556 |
| (39) Texture-GLZSM-Large Zone / High Gray Emphasis | 0.007043928 | 0.083928110 |
| (40) Texture-GLZSM-Gray-Level Non-Uniformity | 0.017761583 | 0.133272588 |
| (41) Texture-GLZSM-Zone Size Non-Uniformity | 0.014020802 | 0.118409468 |
| (42) Texture-GLZSM-Zone Size Percentage | 0.029751166 | 0.172485263 |
| Wavelet-LLL - Histogram - Energy | 0.025387676 | 0.159335106 |
| Wavelet-LLH - Histogram - Variance | 0.023854756 | 0.154449848 |
| Wavelet-LLH - Histogram - Entropy | 0.033415571 | 0.182799266 |
| Wavelet-LHL - Histogram - Variance | 0.030927882 | 0.175863248 |
| Wavelet-LHL - Histogram - Energy | 0.057767441 | 0.240348582 |
| Wavelet-LHL - Histogram - Entropy | 0.050099225 | 0.223828561 |
| Wavelet-LHH - Histogram - Variance | 0.020282252 | 0.142415771 |
| Wavelet-LHH - Histogram - Kurtosis | 0.033793735 | 0.183830724 |
| Wavelet-HLL - Histogram - Variance | 0.015186142 | 0.123232067 |
| Wavelet-HLH - Histogram - Variance | 0.013307481 | 0.115358055 |
| Wavelet-HHL - Histogram - Mean | 0.027852023 | 0.166889254 |
| Wavelet-HHL - Histogram - Skewness | 0.010897238 | 0.104389839 |
| Wavelet-HHL - Histogram - Energy | 0.029348155 | 0.171313033 |
| Hog-1_0_0_0 | 0.014308797 | 0.119619385 |
| Hog-2_0_0_0 | 0.006782632 | 0.082356736 |
| Hog-0_1_0_0 | 0.020070905 | 0.141671822 |
| Hog-2_1_0_0 | 0.032807361 | 0.181128024 |
| Hog-0_2_0_0 | 0.013849870 | 0.117685469 |
| Hog-1_2_0_0 | 0.050361602 | 0.224413907 |
| Hog-0_0_1_0 | 0.016128039 | 0.126996218 |
| Hog-1_1_1_0 | 0.012991240 | 0.113979119 |
| Hog-2_0_2_0 | 0.027158021 | 0.164796908 |
| Hog-1_1_2_0 | 0.014867880 | 0.121933917 |
| Hog-2_1_2_0 | 0.032453580 | 0.180148771 |
| Hog-0_2_2_0 | 0.014063352 | 0.118589003 |
| Hog-1_2_2_0 | 0.022842201 | 0.151136366 |
| Hog-1_0_0_1 | 0.027265867 | 0.165123794 |
| Hog-0_1_0_1 | 0.009087973 | 0.095330862 |
| Hog-0_2_0_1 | 0.011901878 | 0.109095727 |
| Hog-0_0_2_1 | 0.028541484 | 0.168942250 |
| Hog-1_0_2_1 | 0.027350444 | 0.165379697 |
| Hog-2_0_2_1 | 0.033679811 | 0.183520601 |
| Hog-2_1_2_1 | 0.024699502 | 0.157160752 |
| Hog-2_2_2_1 | 0.024918210 | 0.157855030 |
| Hog-1_0_0_2 | 0.023886769 | 0.154553450 |
| Hog-1_1_0_2 | 0.016915256 | 0.130058662 |
| Hog-0_2_0_2 | 0.010699319 | 0.103437515 |
| Hog-1_2_0_2 | 0.039496213 | 0.198736540 |
| Hog-2_2_0_2 | 0.025546602 | 0.159833046 |
| Hog-1_2_1_2 | 0.014355563 | 0.119814703 |
| Hog-2_1_2_2 | 0.021398085 | 0.146280843 |
| Hog-0_2_2_2 | 0.011684197 | 0.108093466 |
| Hog-1_0_0_3 | 0.020255442 | 0.142321615 |
| Hog-0_1_0_3 | 0.020705279 | 0.143893289 |
| Hog-2_1_0_3 | 0.022599053 | 0.150329814 |
| Hog-0_2_0_3 | 0.013168286 | 0.114753154 |
| Hog-0_0_1_3 | 0.016258634 | 0.127509350 |
| Hog-1_0_1_3 | 0.027698093 | 0.166427440 |
| Hog-2_1_1_3 | 0.020937658 | 0.144698508 |
| Hog-1_2_1_3 | 0.019847903 | 0.140882584 |
| Hog-0_0_2_3 | 0.026742813 | 0.163532301 |
| Hog-0_1_2_3 | 0.016901533 | 0.130005895 |
| Hog-1_1_2_3 | 0.029717296 | 0.172387053 |
| Hog-2_1_2_3 | 0.012554337 | 0.112046139 |
| Hog-0_2_2_3 | 0.017317309 | 0.131595248 |
| Hog-2_2_2_3 | 0.031826513 | 0.178399867 |
| Hog-0_0_0_4 | 0.013416610 | 0.115830093 |
| Hog-0_1_0_4 | 0.014842961 | 0.121831692 |
| Hog-1_1_0_4 | 0.007493653 | 0.086565888 |
| Hog-2_1_0_4 | 0.015969913 | 0.126372120 |
| Hog-0_2_0_4 | 0.009228420 | 0.096064664 |
| Hog-1_2_2_4 | 0.027382354 | 0.165476142 |
| Hog-0_0_0_5 | 0.013219048 | 0.114974117 |
| Hog-0_2_0_5 | 0.013575122 | 0.116512326 |
| Hog-0_0_1_5 | 0.009801974 | 0.099004920 |
| Hog-0_1_2_5 | 0.017075144 | 0.130671895 |
| Hog-2_1_2_5 | 0.032537086 | 0.180380392 |
| Hog-0_2_2_5 | 0.029620926 | 0.172107311 |
| Hog-2_0_0_6 | 0.017789177 | 0.133376072 |
| Hog-2_0_1_6 | 0.028383182 | 0.168473089 |
| Hog-0_2_1_6 | 0.032838041 | 0.181212696 |
| Hog-2_0_2_6 | 0.026540409 | 0.162912273 |
| Hog-0_1_2_6 | 0.049765871 | 0.223082655 |
| Hog-0_2_2_6 | 0.012046194 | 0.109755155 |
| Hog-2_2_2_6 | 0.008710792 | 0.093331626 |
| Hog-1_2_1_7 | 0.014863652 | 0.121916576 |
| Hog-0_0_2_7 | 0.015709577 | 0.125337852 |
| Hog-1_0_2_7 | 0.028666830 | 0.169312817 |
| Hog-0_2_2_7 | 0.009435655 | 0.097137298 |
| Hog-2_2_2_7 | 0.012959432 | 0.113839500 |
| Hog-1_0_0_8 | 0.028192521 | 0.167906286 |
| Hog-0_1_0_8 | 0.012909360 | 0.113619363 |
| Hog-2_1_0_8 | 0.017559968 | 0.132514030 |
| Hog-0_2_0_8 | 0.013243551 | 0.115080627 |
| Hog-1_2_0_8 | 0.024756492 | 0.157341958 |
| Hog-0_0_1_8 | 0.013297007 | 0.115312647 |
| Hog-1_0_1_8 | 0.032010353 | 0.178914372 |
| Hog-0_1_1_8 | 0.012704424 | 0.112713904 |
| Hog-1_1_1_8 | 0.013424520 | 0.115864233 |
| Hog-2_1_1_8 | 0.028700864 | 0.169413294 |
| Hog-2_2_1_8 | 0.024617944 | 0.156901064 |
| Hog-1_0_2_8 | 0.020137102 | 0.141905256 |
| Hog-1_1_2_8 | 0.015209535 | 0.123326943 |
| Hog-2_1_2_8 | 0.022030369 | 0.148426307 |
| Hog-0_2_2_8 | 0.015441363 | 0.124263282 |
| Hog-1_2_2_8 | 0.023716034 | 0.154000110 |
| Hog-2_2_2_8 | 0.019351267 | 0.139108832 |
| Hog-1_0_0_9 | 0.016212153 | 0.127326954 |
| Hog-0_1_0_9 | 0.021006482 | 0.144936132 |
| Hog-1_1_0_9 | 0.025473926 | 0.159605532 |
| Hog-2_1_0_9 | 0.025125703 | 0.158510892 |
| Hog-1_2_0_9 | 0.018551607 | 0.136204285 |
| Hog-0_1_1_9 | 0.031824683 | 0.178394738 |
| Hog-1_1_1_9 | 0.025762648 | 0.160507470 |
| Hog-2_1_1_9 | 0.021355285 | 0.146134475 |
| Hog-1_0_2_9 | 0.015908307 | 0.126128139 |
| Hog-2_0_2_9 | 0.013459462 | 0.116014920 |
| Hog-0_1_2_9 | 0.014784325 | 0.121590809 |
| Hog-2_1_2_9 | 0.027953917 | 0.167194249 |
| Hog-1_2_2_9 | 0.014822789 | 0.121748876 |
| Hog-0_0_0_10 | 0.015570540 | 0.124781968 |
| Hog-1_0_0_10 | 0.015243767 | 0.123465653 |
| Hog-2_0_0_10 | 0.007996077 | 0.089420784 |
| Hog-1_1_0_10 | 0.026608622 | 0.163121493 |
| Hog-2_1_0_10 | 0.018554219 | 0.136213871 |
| Hog-1_2_0_10 | 0.044355339 | 0.210607072 |
| Hog-0_0_1_10 | 0.017440593 | 0.132062837 |
| Hog-1_1_1_10 | 0.022153993 | 0.148842175 |
| Hog-2_2_1_10 | 0.039836331 | 0.199590409 |
| Hog-0_0_2_10 | 0.009283873 | 0.096352857 |
| Hog-2_1_2_10 | 0.009730350 | 0.098642536 |
| Hog-1_2_2_10 | 0.028229742 | 0.168017087 |
| Hog-1_0_0_11 | 0.018587811 | 0.136337121 |
| Hog-0_1_0_11 | 0.013361012 | 0.115589846 |
| Hog-2_1_0_11 | 0.022622562 | 0.150407985 |
| Hog-1_0_1_11 | 0.021470122 | 0.146526865 |
| Hog-0_1_1_11 | 0.020232625 | 0.142241433 |
| Hog-2_1_1_11 | 0.020289468 | 0.142441102 |
| Hog-0_0_2_11 | 0.018020820 | 0.134241648 |
| Hog-2_0_2_11 | 0.022344369 | 0.149480329 |
| Hog-0_1_2_11 | 0.017892127 | 0.133761456 |
| Hog-2_1_2_11 | 0.038849126 | 0.197101815 |
| Hog-0_2_2_11 | 0.023488633 | 0.153260016 |
| Hog-2_2_2_11 | 0.012764875 | 0.112981745 |
| Hog-0_1_0_12 | 0.024539414 | 0.156650610 |
| Hog-1_1_0_12 | 0.024859441 | 0.157668771 |
| Hog-2_1_0_12 | 0.035546541 | 0.188537903 |
| Hog-0_2_0_12 | 0.015336068 | 0.123838879 |
| Hog-1_2_0_12 | 0.035535898 | 0.188509676 |
| Hog-2_2_0_12 | 0.011788418 | 0.108574483 |
| Hog-0_1_1_12 | 0.020497662 | 0.143170047 |
| Hog-0_0_2_12 | 0.014980125 | 0.122393321 |
| Hog-1_0_2_12 | 0.018428356 | 0.135751081 |
| Hog-0_1_2_12 | 0.021489529 | 0.146593073 |
| Hog-0_0_0_13 | 0.012611610 | 0.112301423 |
| Hog-1_1_0_13 | 0.022309836 | 0.149364775 |
| Hog-1_0_1_13 | 0.026706938 | 0.163422574 |
| Hog-2_0_1_13 | 0.027968506 | 0.167237871 |
| Hog-1_2_1_13 | 0.025750273 | 0.160468916 |
| Hog-0_1_2_13 | 0.016938520 | 0.130148069 |
| Hog-1_1_2_13 | 0.026602688 | 0.163103305 |
| Hog-2_2_2_13 | 0.027461095 | 0.165713894 |
| Hog-0_1_0_14 | 0.018346829 | 0.135450467 |
| Hog-1_1_0_14 | 0.023684489 | 0.153897657 |
| Hog-2_1_0_14 | 0.023501806 | 0.153302989 |
| Hog-2_2_0_14 | 0.010922287 | 0.104509745 |
| Hog-0_0_2_14 | 0.012480752 | 0.111717286 |
| Hog-0_1_2_14 | 0.015394703 | 0.124075394 |
| Hog-0_1_0_15 | 0.008744357 | 0.093511268 |
| Hog-2_2_0_15 | 0.021123664 | 0.145339824 |
| Hog-0_0_1_15 | 0.014782267 | 0.121582346 |
| Hog-1_0_1_15 | 0.014382325 | 0.119926332 |
| Hog-0_1_1_15 | 0.020254535 | 0.142318429 |
| Hog-2_1_0_16 | 0.035199516 | 0.187615339 |
| Hog-0_0_1_16 | 0.012610638 | 0.112297098 |
| Hog-1_2_1_16 | 0.018843711 | 0.137272397 |
| Hog-0_0_2_16 | 0.013943019 | 0.118080560 |
| Hog-1_0_2_16 | 0.015394560 | 0.124074815 |
| Hog-2_0_2_16 | 0.026831854 | 0.163804317 |
| Hog-1_2_2_16 | 0.011783471 | 0.108551698 |
| Hog-2_2_2_16 | 0.009294769 | 0.096409383 |
| Hog-1_2_1_17 | 0.017031144 | 0.130503426 |
| Hog-2_2_1_17 | 0.009869233 | 0.099344014 |
| Hog-2_2_2_17 | 0.013175031 | 0.114782538 |
| Hog-1_0_0_18 | 0.015290809 | 0.123656011 |
| Hog-2_0_0_18 | 0.026315505 | 0.162220543 |
| Hog-1_1_0_18 | 0.014577588 | 0.120737681 |
| Hog-2_1_0_18 | 0.013760121 | 0.117303544 |
| Hog-1_2_0_18 | 0.020567540 | 0.143413877 |
| Hog-2_2_0_18 | 0.010475984 | 0.102352254 |
| Hog-1_0_1_18 | 0.023291882 | 0.152616781 |
| Hog-1_1_1_18 | 0.014031053 | 0.118452746 |
| Hog-2_1_1_18 | 0.024350878 | 0.156047679 |
| Hog-1_2_1_18 | 0.027868386 | 0.166938270 |
| Hog-2_2_1_18 | 0.037426254 | 0.193458664 |
| Hog-0_0_2_18 | 0.010319760 | 0.101586219 |
| Hog-1_0_2_18 | 0.018058113 | 0.134380479 |
| Hog-1_1_2_18 | 0.020018638 | 0.141487237 |
| Hog-2_1_2_18 | 0.028418406 | 0.168577596 |
| Hog-1_2_2_18 | 0.008291839 | 0.091059535 |
| Hog-1_0_0_19 | 0.019092503 | 0.138175624 |
| Hog-0_1_0_19 | 0.011805714 | 0.108654103 |
| Hog-2_1_0_19 | 0.043398907 | 0.208324043 |
| Hog-1_2_0_19 | 0.044254447 | 0.210367410 |
| Hog-2_0_1_19 | 0.020915354 | 0.144621417 |
| Hog-0_1_1_19 | 0.018096980 | 0.134525016 |
| Hog-1_1_1_19 | 0.024802300 | 0.157487459 |
| Hog-2_1_1_19 | 0.037208287 | 0.192894497 |
| Hog-1_2_1_19 | 0.025835904 | 0.160735511 |
| Hog-2_2_1_19 | 0.022887145 | 0.151284981 |
| Hog-1_0_2_19 | 0.028194664 | 0.167912667 |
| Hog-1_1_2_19 | 0.015508127 | 0.124531629 |
| Hog-2_1_2_19 | 0.041946640 | 0.204808788 |
| Statistics-[0, 1, 0]-Inverse Variance | 0.036863469 | 0.191998617 |
| Statistics-[0, 1, -1]-Inverse Variance | 0.035317551 | 0.187929643 |
| Statistics-[0, 0, -1]-Inverse Variance | 0.022451900 | 0.149839583 |
| Statistics-[0, -1, -1]-Inverse Variance | 0.037302719 | 0.193139119 |
| Statistics-[-1, -1, -1]-Inverse Variance | 0.033313082 | 0.182518716 |
| (2) Texture-Histogram-Variance | 0.024234415 | 0.155674067 |
| (5) Texture-Histogram-Energy | 0.034726188 | 0.186349640 |
| (6) Texture-Histogram-Entropy | 0.037444292 | 0.193505277 |
| (14) Texture-GTSDM-Sum Entropy | 0.035362093 | 0.188048113 |
| (17) Texture-GTSDM-Difference Entropy | 0.033142026 | 0.182049515 |
| (32) Texture-GLZSM-Small Zone Size Emphasis | 0.032152006 | 0.179309803 |
| (40) Texture-GLZSM-Gray-Level Non-Uniformity | 0.012307719 | 0.110940161 |
| (41) Texture-GLZSM-Zone Size Non-Uniformity | 0.013068843 | 0.114319039 |
| (42) Texture-GLZSM-Zone Size Percentage | 0.029794903 | 0.172612002 |
| Wavelet-LLL - Histogram - Variance | 0.037533155 | 0.193734755 |
| Wavelet-LLH - Histogram - Kurtosis | 0.038569774 | 0.196391890 |
| Wavelet-LHL - Histogram - Variance | 0.028411493 | 0.168557092 |
| Wavelet-LHL - Histogram - Energy | 0.033468794 | 0.182944785 |
| Wavelet-LHL - Histogram - Entropy | 0.040934411 | 0.202322542 |
| Wavelet-LHH - Histogram - Variance | 0.020677402 | 0.143796392 |
| Wavelet-HLL - Histogram - Variance | 0.017647716 | 0.132844706 |
| Wavelet-HLL - Histogram - Energy | 0.042382626 | 0.205870410 |
| Wavelet-HLL - Histogram - Entropy | 0.032517438 | 0.180325922 |
| Wavelet-HLH - Histogram - Variance | 0.016247152 | 0.127464315 |
| Wavelet-HLH - Histogram - Entropy | 0.034455425 | 0.185621725 |
| Wavelet-HHL - Histogram - Variance | 0.023526009 | 0.153381907 |
| Wavelet-HHL - Histogram - Energy | 0.046466900 | 0.215561825 |
| Wavelet-HHL - Histogram - Entropy | 0.038202634 | 0.195454941 |
| Wavelet-HHH - Histogram - Variance | 0.030900472 | 0.175785301 |
| Hog-2_0_0_0 | 0.013280630 | 0.115241616 |
| Hog-0_2_0_0 | 0.033473398 | 0.182957366 |
| Hog-2_0_1_0 | 0.032521362 | 0.180336802 |
| Hog-2_1_1_0 | 0.026662241 | 0.163285763 |
| Hog-1_2_2_0 | 0.031298770 | 0.176914584 |
| Hog-2_0_0_1 | 0.032409433 | 0.180026201 |
| Hog-2_1_0_1 | 0.021202802 | 0.145611818 |
| Hog-2_0_1_1 | 0.031926435 | 0.178679700 |
| Hog-2_1_1_1 | 0.027180241 | 0.164864311 |
| Hog-1_2_0_2 | 0.009576272 | 0.097858429 |
| Hog-1_2_2_2 | 0.014905179 | 0.122086770 |
| Hog-1_1_0_3 | 0.017456643 | 0.132123589 |
| Hog-2_1_0_3 | 0.017195195 | 0.131130449 |
| Hog-2_1_2_3 | 0.019919782 | 0.141137457 |
| Hog-2_2_2_3 | 0.023118338 | 0.152047158 |
| Hog-2_0_0_4 | 0.020839202 | 0.144357896 |
| Hog-2_1_0_4 | 0.022614548 | 0.150381342 |
| Hog-1_2_2_4 | 0.030429297 | 0.174439952 |
| Hog-0_0_0_5 | 0.010997201 | 0.104867539 |
| Hog-2_0_0_5 | 0.015556815 | 0.124726963 |
| Hog-0_0_0_6 | 0.019772081 | 0.140613231 |
| Hog-2_0_0_6 | 0.022166090 | 0.148882805 |
| Hog-0_0_1_6 | 0.025079110 | 0.158363852 |
| Hog-2_0_1_6 | 0.010952525 | 0.104654314 |
| Hog-0_2_0_7 | 0.008986906 | 0.094799292 |
| Hog-0_2_1_7 | 0.014105779 | 0.118767751 |
| Hog-2_1_2_7 | 0.029553823 | 0.171912253 |
| Hog-0_1_0_8 | 0.012398199 | 0.111347202 |
| Hog-2_1_0_8 | 0.033705694 | 0.183591106 |
| Hog-0_2_0_8 | 0.009039976 | 0.095078790 |
| Hog-1_2_0_8 | 0.021824176 | 0.147730080 |
| Hog-1_0_1_8 | 0.016087981 | 0.126838405 |
| Hog-2_0_1_8 | 0.021687729 | 0.147267543 |
| Hog-1_2_1_8 | 0.041783331 | 0.204409713 |
| Hog-1_1_2_8 | 0.012231427 | 0.110595780 |
| Hog-1_0_0_9 | 0.012598269 | 0.112242009 |
| Hog-2_2_0_9 | 0.023664086 | 0.153831355 |
| Hog-2_0_1_9 | 0.033474002 | 0.182959018 |
| Hog-2_1_1_9 | 0.013406338 | 0.115785741 |
| Hog-1_2_1_9 | 0.012967334 | 0.113874203 |
| Hog-0_0_2_9 | 0.008468126 | 0.092022418 |
| Hog-1_1_2_9 | 0.010355480 | 0.101761877 |
| Hog-2_1_2_9 | 0.041119093 | 0.202778434 |
| Hog-0_0_0_10 | 0.013322402 | 0.115422711 |
| Hog-0_0_1_10 | 0.009910233 | 0.099550154 |
| Hog-2_0_1_10 | 0.036642288 | 0.191421754 |
| Hog-0_0_2_10 | 0.013863595 | 0.117743766 |
| Hog-0_2_2_10 | 0.011491567 | 0.107198725 |
| Hog-2_0_0_11 | 0.020289666 | 0.142441798 |
| Hog-0_1_0_11 | 0.014004162 | 0.118339184 |
| Hog-2_0_2_11 | 0.015894973 | 0.126075269 |
| Hog-2_1_0_12 | 0.022529720 | 0.150099034 |
| Hog-2_2_0_12 | 0.018677580 | 0.136665943 |
| Hog-2_1_1_12 | 0.012613197 | 0.112308491 |
| Hog-1_1_2_12 | 0.011665963 | 0.108009089 |
| Hog-0_2_2_12 | 0.010977519 | 0.104773654 |
| Hog-0_0_0_13 | 0.007570087 | 0.087006249 |
| Hog-0_1_0_13 | 0.009177297 | 0.095798208 |
| Hog-1_1_0_13 | 0.024289512 | 0.155850930 |
| Hog-0_2_0_13 | 0.016808928 | 0.129649250 |
| Hog-2_1_1_13 | 0.015960315 | 0.126334140 |
| Hog-0_0_2_13 | 0.011894537 | 0.109062079 |
| Hog-0_1_2_13 | 0.039109831 | 0.197762057 |
| Hog-1_1_2_13 | 0.020254370 | 0.142317847 |
| Hog-2_1_2_13 | 0.018140486 | 0.134686622 |
| Hog-2_2_2_13 | 0.018677957 | 0.136667324 |
| Hog-2_1_0_14 | 0.025580010 | 0.159937518 |
| Hog-2_2_0_14 | 0.022761048 | 0.150867652 |
| Hog-0_0_2_14 | 0.024075847 | 0.155163937 |
| Hog-0_1_2_14 | 0.015612060 | 0.124948231 |
| Hog-0_2_2_14 | 0.023489718 | 0.153263556 |
| Hog-2_0_0_15 | 0.029577149 | 0.171980083 |
| Hog-0_0_2_15 | 0.015995714 | 0.126474162 |
| Hog-0_1_2_15 | 0.017146403 | 0.130944274 |
| Hog-0_2_2_15 | 0.013859139 | 0.117724845 |
| Hog-0_0_0_16 | 0.020716259 | 0.143931438 |
| Hog-0_2_0_16 | 0.009053806 | 0.095151491 |
| Hog-0_0_1_16 | 0.022014852 | 0.148374026 |
| Hog-2_0_1_16 | 0.023111316 | 0.152024063 |
| Hog-0_0_0_18 | 0.010252899 | 0.101256598 |
| Hog-1_0_0_18 | 0.023002445 | 0.151665570 |
| Hog-2_1_0_18 | 0.028886301 | 0.169959704 |
| Hog-0_2_0_18 | 0.025439482 | 0.159497593 |
| Hog-1_2_0_18 | 0.016532165 | 0.128577468 |
| Hog-2_2_0_18 | 0.030042840 | 0.173328704 |
| Hog-1_0_1_18 | 0.014223111 | 0.119260684 |
| Hog-2_0_1_18 | 0.017170309 | 0.131035525 |
| Hog-1_1_1_18 | 0.019003905 | 0.137854652 |
| Hog-2_1_1_18 | 0.033412942 | 0.182792072 |
| Hog-0_2_1_18 | 0.024618446 | 0.156902663 |
| Hog-0_0_2_18 | 0.015477541 | 0.124408764 |
| Hog-2_0_2_18 | 0.023530689 | 0.153397159 |
| Hog-1_1_2_18 | 0.021088906 | 0.145220199 |
| Hog-2_1_2_18 | 0.035659931 | 0.188838372 |
| Hog-0_1_0_19 | 0.017324880 | 0.131624010 |
| Hog-2_1_0_19 | 0.022377888 | 0.149592405 |
| Hog-1_2_0_19 | 0.015362079 | 0.123943852 |
| Hog-1_0_1_19 | 0.020032760 | 0.141537133 |
| Hog-2_0_1_19 | 0.036880281 | 0.192042395 |
| Hog-1_1_1_19 | 0.014337759 | 0.119740382 |
| Hog-1_1_2_19 | 0.017229265 | 0.131260295 |
| Statistics-[0, 1, 0]-Contrast | 0.039933347 | 0.199833297 |
| Statistics-[0, 1, 0]-Variance | 0.037737714 | 0.194261972 |
| Statistics-[0, 1, 0]-Inertia | 0.039933347 | 0.199833297 |
| Statistics-[0, 1, 0]-Inverse Variance | 0.049468177 | 0.222414426 |
| Statistics-[-1, 1, 0]-Contrast | 0.039184367 | 0.197950415 |
| Statistics-[-1, 1, 0]-Variance | 0.037055343 | 0.192497645 |
| Statistics-[-1, 1, 0]-Inertia | 0.039184367 | 0.197950415 |
| Statistics-[-1, 0, 0]-Variance | 0.039531835 | 0.198826142 |
| Statistics-[-1, 0, 0]-Cluster Tendency | 0.025528659 | 0.159776905 |
| Statistics-[-1, -1, 0]-Contrast | 0.036299026 | 0.190523033 |
| Statistics-[-1, -1, 0]-Variance | 0.037030107 | 0.192432083 |
| Statistics-[-1, -1, 0]-Inertia | 0.036299026 | 0.190523033 |
| Statistics-[0, 1, -1]-Contrast | 0.037287599 | 0.193099971 |
| Statistics-[0, 1, -1]-Variance | 0.037449955 | 0.193519910 |
| Statistics-[0, 1, -1]-Inertia | 0.037287599 | 0.193099971 |
| Statistics-[0, 1, -1]-Inverse Variance | 0.048108805 | 0.219337195 |
| Statistics-[0, 0, -1]-Entropy | 0.036738452 | 0.191672772 |
| Statistics-[0, 0, -1]-Contrast | 0.029275975 | 0.171102237 |
| Statistics-[0, 0, -1]-Variance | 0.040026041 | 0.200065093 |
| Statistics-[0, 0, -1]-Inertia | 0.029275975 | 0.171102237 |
| Statistics-[0, 0, -1]-Cluster Tendency | 0.023527892 | 0.153388044 |
| Statistics-[0, 0, -1]-Inverse Variance | 0.050709946 | 0.225188691 |
| Statistics-[0, -1, -1]-Contrast | 0.038817851 | 0.197022463 |
| Statistics-[0, -1, -1]-Variance | 0.037506184 | 0.193665133 |
| Statistics-[0, -1, -1]-Inertia | 0.038817851 | 0.197022463 |
| Statistics-[0, -1, -1]-Inverse Variance | 0.048922719 | 0.221184806 |
| Statistics-[-1, 0, -1]-Variance | 0.039236137 | 0.198081138 |
| Statistics-[-1, 0, -1]-Cluster Tendency | 0.025309687 | 0.159090184 |
| Statistics-[1, 0, -1]-Variance | 0.039323132 | 0.198300610 |
| Statistics-[1, 0, -1]-Cluster Tendency | 0.024030347 | 0.155017247 |
| Statistics-[-1, 1, -1]-Contrast | 0.037241588 | 0.192980797 |
| Statistics-[-1, 1, -1]-Variance | 0.036732491 | 0.191657222 |
| Statistics-[-1, 1, -1]-Inertia | 0.037241588 | 0.192980797 |
| Statistics-[1, -1, -1]-Contrast | 0.037315724 | 0.193172783 |
| Statistics-[1, -1, -1]-Variance | 0.036874502 | 0.192027346 |
| Statistics-[1, -1, -1]-Inertia | 0.037315724 | 0.193172783 |
| Statistics-[-1, -1, -1]-Contrast | 0.034991825 | 0.187061020 |
| Statistics-[-1, -1, -1]-Variance | 0.036763230 | 0.191737400 |
| Statistics-[-1, -1, -1]-Inertia | 0.034991825 | 0.187061020 |
| Statistics-[1, 1, -1]-Contrast | 0.037784845 | 0.194383243 |
| Statistics-[1, 1, -1]-Variance | 0.036791687 | 0.191811592 |
| Statistics-[1, 1, -1]-Inertia | 0.037784845 | 0.194383243 |

Abbreviations: MRI, magnetic resonance imaging; GLZSM, grey level zone size matrix; Hog, histogram of oriented gradient; GTSDM, grey tone spatial dependence matrix.
